# Patient-Specific Assays Based on Whole-Genome Sequencing Data to Measure Residual Disease in Children With Acute Lymphoblastic Leukemia: A Proof of Concept Study

**DOI:** 10.3389/fonc.2022.899325

**Published:** 2022-07-05

**Authors:** Cecilia Arthur, Fatemah Rezayee, Nina Mogensen, Leonie Saft, Richard Rosenquist, Magnus Nordenskjöld, Arja Harila-Saari, Emma Tham, Gisela Barbany

**Affiliations:** ^1^ Department of Clinical Genetics, Karolinska University Hospital, Stockholm, Sweden; ^2^ Department of Molecular Medicine and Surgery and Center for Molecular Medicine, Karolinska Institutet, Stockholm, Sweden; ^3^ Department of Pediatric Oncology, Karolinska University Hospital, Stockholm, Sweden; ^4^ Department of Women’s and Children’s Health, Karolinska Institutet, Stockholm, Sweden; ^5^ Department of Clinical Pathology and Cancer Diagnostics, Karolinska University Hospital, Stockholm, Sweden; ^6^ Department of Oncology-Pathology, Karolinska Institutet, Stockholm, Sweden; ^7^ Department of Women’s and Children’s Health, Uppsala University, Uppsala, Sweden

**Keywords:** acute lymphoblastic leukemia, liquid biopsy, disease monitoring, precision medicine, whole-genome sequencing, structural variation, technical feasibility, diagnostic performance

## Abstract

Risk-adapted treatment in acute lymphoblastic leukemia (ALL) relies on genetic information and measurable residual disease (MRD) monitoring. In this proof of concept study, DNA from diagnostic bone marrow (BM) of six children with ALL, without stratifying genetics or central nervous system (CNS) involvement, underwent whole-genome sequencing (WGS) to identify structural variants (SVs) in the leukemic blasts. Unique sequences generated by SVs were targeted with patient-specific droplet digital PCR (ddPCR) assays. Genomic DNA (gDNA) from BM and cell-free DNA (cfDNA) from plasma and cerebrospinal fluid (CSF) were analyzed longitudinally. WGS with 30× coverage enabled target identification in all cases. Limit of quantifiability (LoQ) and limit of detection (LoD) for the ddPCR assays (n = 15) were up to 10^−5^ and 10^−6^, respectively. All targets were readily detectable in a multiplexed ddPCR with minimal DNA input (1 ng of gDNA) at a 10^−1^ dilution, and targets for half of the patients were also detectable at a 10^−2^ dilution. The level of MRD in BM at end of induction and end of consolidation block 1 was in a comparable range between ddPCR and clinical routine methods for samples with detectable residual disease, although our approach consistently detected higher MRD values for patients with B-cell precursor ALL. Additionally, several samples with undetectable MRD by flow cytometry were MRD-positive by ddPCR. In plasma, the level of leukemic targets decreased in cfDNA over time following the MRD level detected in BM. cfDNA was successfully extracted from all diagnostic CSF samples (n = 6), and leukemic targets were detected in half of these. The results suggest that our approach to design molecular assays, together with ddPCR quantification, is a technically feasible option for accurate MRD quantification and that cfDNA may contribute valuable information regarding MRD and low-grade CNS involvement.

## Introduction

The outcome of acute lymphoblastic leukemia (ALL), the most common form of childhood leukemia, has improved dramatically over the last decades. A key factor to this success has been the implementation of risk-adapted treatment intensity, based on biological and genetic factors as well as on response to induction treatment (1). The genetic aberrations of the leukemic cells contribute important information for the risk stratification of ALL patients. Consequently, modern protocols require an extensive genetic characterization of the blasts at diagnosis to correctly stratify the patients to distinct genetic risk categories. This is a labor-intensive process due to the heterogeneous nature of the genetic aberrations, requiring a range of methods for each patient.

The persistence of leukemic cells in the bone marrow (BM) after induction treatment, so-called measurable residual disease (MRD), is the most important prognostic factor in ALL (2, 3). The detection of leukemic blasts by flow cytometry (FCM) is based on the quantification of cells with leukemia-associated immunophenotype (LAIP), while molecular techniques, such as real-time quantitative PCR (RQ-PCR), rely on the detection and quantification of the clonal immunoglobulin and T-cell receptor (IG/TR) gene rearrangements (4, 5). Because of FCM’s short turnover time, the use of this method is widespread, but not all patients have LAIPs that allow for accurate and sensitive MRD detection. Utilizing IG/TR gene rearrangements to measure MRD has been a gold standard approach in many treatment protocols (5). The method, however, is demanding and requires expert laboratories to achieve the desired reproducibility and sensitivity (6). Thus, it is challenging to allocate the patients to the genetic risk categories and provide all patients with a precision marker for MRD monitoring with the currently used methods.

Central nervous system (CNS) involvement is detected in 3%–10% of ALL patients at diagnosis; however, the CNS may act as a sanctuary for leukemic cells, and all patients are given prophylactic intrathecal treatment (7, 8). If CNS involvement is demonstrated, treatment is intensified; hence, morphologic examination of cerebrospinal fluid (CSF) is mandatory in ALL.

High-throughput sequencing technologies have dramatically expanded our knowledge regarding the genetic events that drive leukemic transformation and whole-genome sequencing (WGS), and transcriptome profiling has successfully identified putative drivers in the majority of patients (9). Also, WGS has shown promising potential in the diagnostic setting of hematological malignancies and detects clinically relevant genomic aberrations beyond the resolution of conventional methods (10–15).

Cell-free DNA (cfDNA), and more specifically cell-free tumor DNA (ctDNA), has shown promise as a biomarker for cancer in liquid biopsies, yet only a handful of studies have explored the potential role of plasma DNA as a marker for MRD quantification in acute leukemias. Schwartz and coworkers showed that in ALL the elevation of total cfDNA in plasma, without differentiating between ctDNA and normal cfDNA, could potentially be used as an MRD marker. The same study also showed that quantitative analysis of IG/TR gene rearrangements demonstrated high concordance between plasma and peripheral blood (PB) leukocytes (16). Another study on pediatric ALL investigated the clonal IG/TR gene rearrangements in plasma and compared the level to MRD assessed in BM by FCM. The methods showed poor correlation, and yet the authors concluded that both approaches could independently predict relapse (17).

In this proof-of-principle study, we challenge WGS to detect genomic aberrations in pediatric ALL and use the novel sequences, created by the structural rearrangement junction sites, as leukemia-specific molecular targets. Patient-specific droplet digital PCR (ddPCR) assays directed toward these targets were designed and used to measure the level of disease in BM samples taken at diagnosis and follow-up, as well as to investigate the presence of these targets in the cfDNA shed into the plasma and CSF before and during therapy. Finally, results were compared to MRD findings from routine methods.

## Materials and Methods

### Patients and Samples

Four patients with precursor B-cell ALL (BCP-ALL) and two patients with T-ALL were included in the study. All were diagnosed during 2018–2019 and treated according to the Nordic Society for Pediatric Hematology and Oncology (NOPHO) 2008 protocol (18) at Karolinska University Hospital, Stockholm, Sweden. Routine investigations of the diagnostic BM samples included morphology, FCM, chromosome banding analysis (CBA), fluorescence *in situ* hybridization (FISH), and array-based comparative genomic hybridization (aCGH). Clinical data were retrieved from medical records. None of the BCP-ALL patients had intermediate risk (IR) or high risk (HR) cytogenetic aberrations. Three of these were classified as “B-other” and one as high hyperdiploid, and thus they were classified as having standard risk genetics in the NOPHO 2008 ALL protocol. One patient (patient 4, B-other) suffered from two relapses. Patients 5 and 6 were included to test the approach in T-ALL. **Table 1** summarizes the patients’ characteristics at diagnosis.

**Table 1:** Patient characteristics.

UPN	IP/genetic classification	Genetic findings	Age (years)	% blasts bm d0	WBCC (×10^9^/L) d0	Final stratification	Status at last follow-up
1	BCP-ALL/B-other	46,XX	5	65	6.5	IR	CR1
		arr[hg19] 3p21.31(46,559,773_48,128,417)x1					
		split FISH *TCF3* signal in 53% cells					
2	BCP-ALL/B-other	46,XY	13	93	103	HR	CR1
		arr[hg19]7p12.2(50,418,802_50,482,032)x0,9p21.3(20,977,882_22,463,282)x0,9p13.2(36,891,779_37,327,910)x1,9p13.1p11.1(38,976,057_47,312,257)x3					
3	BCP-ALL/HeH	54,XY,+21,+21,inc	3	80	6.4	SR	CR1
		arr[hg19](X)x4,(4,14,17)x3,2p25.3p24.1x3,6p25.3q16.1x3,6q16.1q22.31x1,8p21.2q21.11x3,(21)x4					
4	BCP-ALL/B-other	46,XX,del(4)(p14),del(9)(p1?3),-14,del(20)(p12),add(21)(q22),+mar[cp17]	11	80	30.4	IR	
		arr[hg19] 4p15.1(30,669,209_31,935,830)x1,4q13.3(71,325,846_ 72,755,788)x1,9p24.1p13.3(5,185,235_34,655,127)x1,9p21.3p21.1(19,894,810_31,454,585)x0,20p12.1p11.23(14,082,197_21,209,724)x1					
	Relapse 1	arr[hg19] 4p15.1(30,669,209_31,935,830)x1,4q13.3(71,325,846_72,755,788)x1,9p24.1p13.3(5,185,235_34,655,127)x1,9p21.3p21.1(19,894,810_31,454,585)x0,(13)x3,20p12.1p11.23(14,082,197_21,209,724)x1	12	90	10.6	HR relapse	
	Relapse 2 (post-SCT)	Identical to relapse 1	13	35	8.5		deceased
5	T-ALL/N.A.	46,XY,t(5;7)(q35;q21),-10,+mar	8	76	249	IR high (A2G)	CR1
		arr[hg19]5p15.32p15.31(4,592,987_7,676,318)x1,7q21.2(92,317,833_92,456,363)x1,8p22p21.3(17,412,016_19,012,864)x1,9p21.3(21,612,192_21,965,674)x1,(21,965,674_21,301,793)x0,(21,301,793_22,502,804)x1,16q22.1(67,418,460_68,680,751)x1					
6	T-ALL/N.A.	46,XY,del(6)(q14q15)	12	88	580	HR	CR1
		arr[hg19]1p33(47,699,244_47,779,355)x1,6q14.1q15(77,236,913_89,600,323)x1,9p21.3(21,883,627_22,052,145)x0					

UPN, unique patient number; IP, immunophenotype; BM, bone marrow; WBCC, white blood cell count; BCP-ALL, B-cell precursor acute lymphoblastic leukemia; B-other, BCP-ALL without any of the routinely assessed classifying genetic aberrations; HeH, high hyperdiploidy (51–65 chromosomes in the leukemic clone); SCT, stem cell transplantation; T-ALL, T-cell acute lymphoblastic leukemia; N.A., not applicable; CR1, first complete remission A2G, ALLTogether protocol.

Blood and CSF samples were collected in Cell-Free DNA BCT tubes (10 ml) (STRECK, La Vista, NE, USA) and stored at room temperature for a maximum of 5 days. Sample volumes were 1–5 ml (plasma) and 0.15–4 ml (CSF). Cell removal was performed by double centrifugation (10 min at 4°C 1,600 ×*g* and 16,000 ×*g*), and supernatants were frozen at −80°C. Plasma from blood donors was processed in the same manner for use as plasma negative control (NC). cfDNA was isolated in a semi-automated fashion using QiAamp Circulating Nucleic Acid Kit™ on the QIAvac24 Plus vacuum manifold and the Qiacube™ (Qiagen, Manchester, UK). cfDNA was eluted in 40 μl of AVE buffer and stored at −20°C for a maximum of 3 months.

Diagnostic BM samples were collected in 5-ml EDTA tubes for routine genetic investigations, and follow-up BM samples (Ficoll-separated mononuclear cells) were retrieved from the Karolinska University Hospital biobank. Genomic DNA (gDNA) was isolated using a Tissue kit on EZ1™ Automated (Qiagen, Hilden, Germany) and stored at −20°C until use. If automated gDNA extraction resulted in a low yield (<30 ng/μl), manual isolation was performed using AllPrep DNA/RNA Mini Kit™ (Qiagen, Hilden, Germany). NC was extracted from PB from healthy blood donors, using QIAsymphony DSP DNA Midi Kit (Qiagen, Hilden, Germany), and anonymized and pooled prior to use.

### Measurable Residual Disease Analysis by Flow Cytometry and Real-Time Quantitative PCR

Immunophenotyping of diagnostic and follow-up BM aspirates was performed on FACSCanto/FACS Lyric flow cytometers according to the NOPHO 2008 (until September 2019) and the ALLTogether (A2G) protocols (19) using standardized MRD 6- and 8-color antibody panels (**Supplementary Table 1**) (all from Becton Dickinson, Franklin Lakes, NJ, USA) and the Infinicyt software program for MRD analysis. Guidelines for sample preparation and data analysis followed the NOPHO and ALLTogether laboratory guidelines. Events with LAIP were identified in all plots of interest. The final MRD value was calculated as the mean percentage of leukemic cells of all living events excluding erythropoiesis. To be considered MRD, a cluster of cells (≥10 events) with LAIP had to be identified. A cluster of >40 events with a leukemic phenotype was considered a quantitative residual disease. If no cluster was detected, the result was termed “not detectable” at a given sensitivity level.

Molecular screening and MRD analysis by RQ-PCR were performed in accordance with EuroMRD guidelines using IG/TR gene rearrangements as targets and consensus primers (5). DNA was extracted from mononuclear BM cells following Ficoll density centrifugation of the BM aspirate using the QIAamp MiniKit according to the manufacturer’s protocol.

### Whole-Genome Sequencing, Variant Calling, and Target Identification

gDNA measuring 1.1 µg from diagnostic BM samples was sequenced using a PCR-free, paired-end WGS protocol with a 30× coverage on an Illumina HiSeqX Ten platform at Clinical Genomics, SciLifeLab, Stockholm, Sweden (20). The Human GRCh37 (hg19) RefSeq (21) was used for annotation. Structural variants (SVs) were identified using MIP (22) and visualized in the SCOUT interface (23). MIP performs SV detection using CNVnator V0.3.2 (24) with 1-kb bins, Delly (25) TIDDIT V2.0.0 (26), and Manta (27). Filtering was performed using a list of recurrent aberrations in ALL (**Supplementary Table 2**). If no such aberration was detected in a sample, the copy number variation (CNV) data from routine aCGH were scrutinized for events likely to result in novel leukemia-specific junction sequences. Candidate regions were then manually inspected in Integrative Genomics Viewer (28, 29). Whenever possible, the junction sites created by SVs involving genes known to be recurrently involved in ALL were chosen as potential targets.

### Droplet Digital PCR Assay Design

Custom ddPCR assays covering SV breakpoint sequences were designed according to the Rare Mutation Detection Best Practices Guidelines (Bio-Rad, Hercules, CA, USA) using Primer3Plus software (30). When junctions were located in regions deemed unsuitable for primer design, manual design was attempted. In-Silico PCR and Human BLAT Search (31) were executed to ensure the specificity of the assays. Primers and probes were purchased from Integrated DNA Technologies™ (IDT, Coralville, IA, USA). Amplicon sizes were <100 bp. All target probes were labeled with 5′ 6-FAM™ fluorophores and 3′ Iowa Black^®^FQ quenchers. Sequence details are available upon request. Assays for reference genes (*ABCC9* or *ALB*) labeled with 5′ HEX fluorophores and 3′ BHQ-1 quenchers were purchased from Bio-Rad IDT, respectively. Assays were prepared with a final primer/probe concentration of 900/250 nM, and ddPCRs were run on the QX200 AutoDG Droplet Digital PCR System/QX200 Droplet Reader (Bio-Rad, CA, USA) according to the manufacturer’s instructions. All reactions contained a reference gene assay (*ALB* or *ABCC9*) to control for DNA concentration and amplifiability.

### Assay Optimization and Performance Testing

The optimal annealing temperature was determined through a gradient-ddPCR ranging from 55°C to 65°C on single and multiplexed assay reactions using diagnostic BM gDNA as the template (positive control (PC)). ddPCRs contained 10 μl of 2× Supermix for Probes (No dUTP) (Bio-Rad, CA, USA), 1 μl of 20× assay mix (primers and probes at 900/450 nM), and 11 μl of gDNA/cfDNA eluate. gDNA input was titrated from 66 to 500 ng, and undigested versus digested gDNA was compared. A no-template control (NTC; nuclease-free water) and an NC were included in each run. Specificity and sensitivity were assessed by running 500 ng of undigested gDNA from serial 10-fold dilutions of PC from each patient in NC (ranging from 10^−1^ to 10^−6^). All assays were individually tested for the limit of detection (LoD) and limit of quantifiability (LoQ). Ultimately, minimal DNA input ddPCRs were performed in a multiplexed manner with 1 ng of gDNA from 10^−1^ and 10^−2^ dilutions (containing 100 and 10 pg of PC, respectively). Thermal cycling conditions were as follows: 1 cycle at 95°C for 10 min, 40 cycles at 94°C for 30 s and (individualized annealing temperature) for 1 min, 1 cycle at 98°C for 10 min, and 1 cycle at 4°C infinite, all at a ramp rate of 2°C/s.

### Quantification of Targets in Patient Samples

For BM sample analysis, 500 ng of undigested gDNA was divided into five ddPCRs. For plasma and CSF sample analysis, 11 μl of cfDNA eluate was loaded in triplicate ddPCRs. Triplicates of controls were run on all plates (NTC, NC, and PC) with the addition of 9–12 wells of plasma NC for cfDNA analysis.

Output data were analyzed using the QuantaSoftPro/QX Manager Software (Bio-Rad, Hercules, CA, USA), and results were manually reviewed. Thresholds for empty (negative), target positive/reference negative, target negative/reference positive, and double-positive droplets were manually set based on the control samples included in each run according to the provider’s instructions. For a sample to be considered target positive, three or more droplets with FAM-fluorescence were required. One to two positive droplets are referred to as trace amounts.

The average copy number (CN) of target and reference molecules (FAM and HEX signals, respectively) per ml of liquid biopsy (plasma or CSF) was calculated by dividing the sum of all signals from the three replicate ddPCRs by initial liquid biopsy volume. The ratio of target molecules to reference molecules in cfDNA and gDNA (plasma and BM, respectively) was calculated after correction for the number of targets for each patient. In cases where BM samples from diagnosis were not available for ddPCR analysis (patients 1, 4, and 5), a theoretical ratio was extrapolated from the dilution series data generated by assay performance testing.

## Results

**Figure 1:**
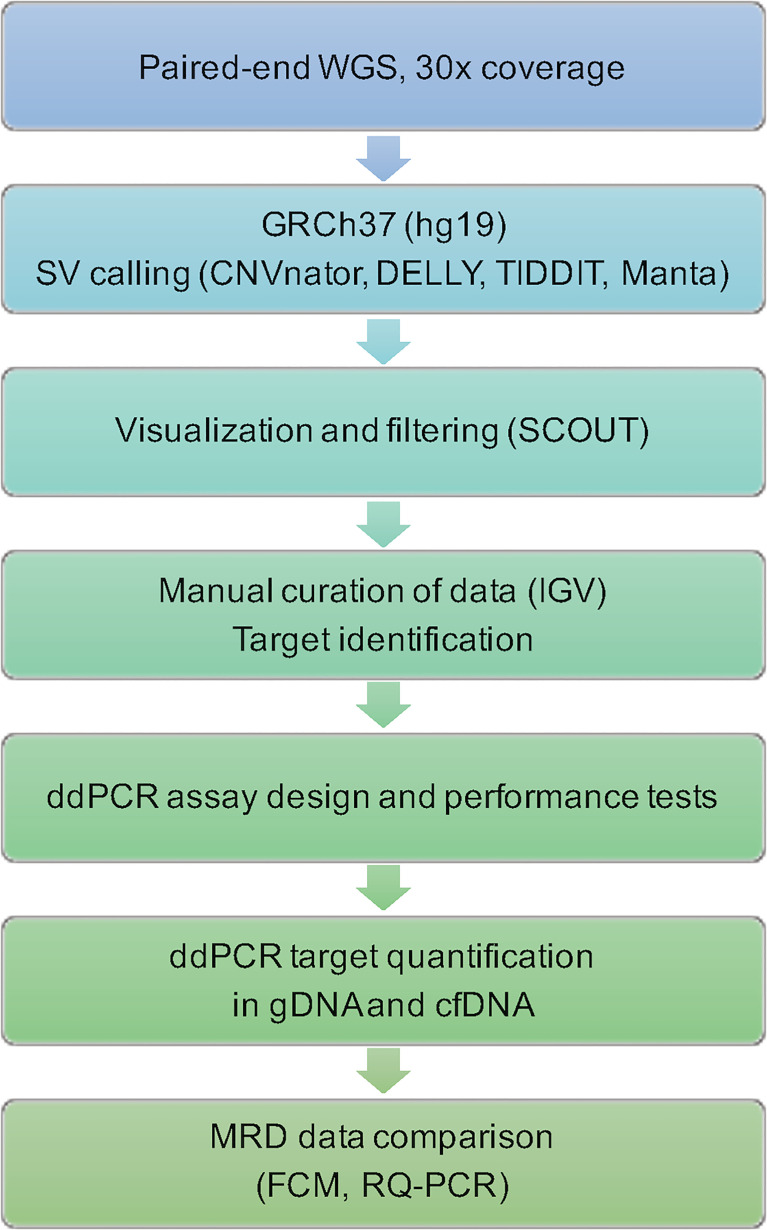
Flowchart of steps and techniques used for identification and quantification of leukemic targets. WGS, whole-genome sequencing; GRCh37, Genome Reference Consortium Human Build 37; SV, structural variant; IGV, The Integrative Genomics Viewer; ddPCR, droplet digital PCR; gDNA, genomic DNA; cfDNA, cell-free DNA; MRD, measurable residual disease; FCM, flow cytometry; RQ-PCR, real-time quantitative PCR.

A schematic flowchart of the techniques used to process WGS data, design personalized assays and analyze the results is provided in **Figure 1**.

### Whole-Genome Sequencing Data Enable Target Identification

Our aim was to identify up to three targets per patient from the WGS data using the following criteria: i) events supported by reads that indicated their presence in a majority of leukemic blasts, ii) unique sequences generated by SVs in the leukemic blasts, and iii) recurrent stratifying genetic events, alternatively putative driving events, described as recurrent in the literature.

**Figure 2:**
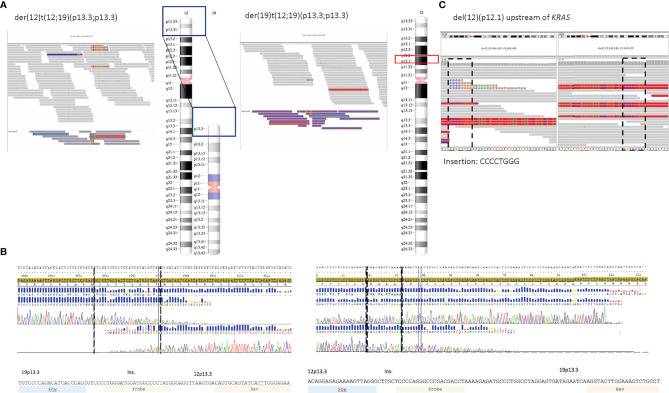
**(A–C)** Target sequences for patient 1. **(A)** Screenshots from IGV showing the junction sequences on derivative chromosomes 12 and 19 and ideogram representing the regions involved in the reciprocal translocation. Discordant reads are marked in medium gray (*ZNF384* locus) and purple (*TCF3* locus). **(B)** Sanger sequencing confirmed the sequence to be identical to the sequence derived from WGS data, including the non-templated insertion in both junctions (sequences within broken lines). **(C)** Screenshots from IGV (discordant reads in red) and ideogram representing the deletion on 12p12.1 (upstream of *KRAS*). IGV, Integrated Genome Viewer; WGS, whole-genome sequencing.

FISH results indicated a split signal pattern for *TCF3* (19p13.3) in 53% of BM cells for patient 1. The WGS data supported the presence of a reciprocal translocation between chromosomes 12 and 19, t(12;19)(p13;p13), and non-templated insertions at the junctions in both derivative chromosomes, which were selected as targets (**Figure 2A**). The rearrangement created a *TCF3*::*ZNF384* fusion, reported as recurrent in ALL (32). The sequence junctions were confirmed with Sanger sequencing (**Figure 2B**). WGS data also revealed a deletion upstream of *KRAS* gene on chromosome 12 (p12.1), including an 8-bp non-templated insertion at the junction site (**Figure 2C**).

WGS results for patient 2 indicated intrachromosomal events on chromosome 9, resulting in deletions affecting both chromosome homologs. Polynucleotide stretches were found at the breakpoint junctions for both deletions. Nonetheless, assays were attempted but failed to amplify specific PCR products. The WGS data detected two additional deletions, both affecting transcription factors essential for lymphocyte development and recurrent events in ALL, *IKZF1* on chromosome 7 (p12.2) (33) and *PAX5* on chromosome 9 (p13.2) (34) (**Figures S1A, B**). The junction sequences resulting from these two deletions were selected as targets.

Routine analysis for patient 3 revealed a high hyperdiploid clone with 54 chromosomes and several CN alterations (CNAs) in 70% of the BM cells. These CNAs including an extra derivative chromosome 8 were supported by the WGS data as well. However, inspection in Integrated Genome Viewer (IGV) showed that most of these events had occurred in highly repetitive regions (**Figure S2A**) and thus were inadequate as targets. Only one suitable SV junction resulting from an unbalanced rearrangement between chromosomes 2 and 8 was detected and was selected as the only target for patient 3. In order to maximize the probability of detecting this target, a double-probe assay was designed in which two (non-overlapping) probes were located over the junction, one on the plus strand and one on the minus strand (**Figure S2B**).

For patient 4, the callers for SVs identified several aberrations affecting chromosome arms 4p, 9p, 20p, and 21q in the WGS data. These events had also been detected by the routine methods and were all retained in both relapses. Out of these, three junction sequences were selected following an inspection in IGV (**Figures S3A–C**). The first target was the junction created by a deletion on chromosome arm 9p, encompassing *CDKN2A/B*. The WGS data indicated a pericentric inversion of chromosome 4, with breakpoints in 4p14 and 4q13.3, resulting in a deletion together with a 6-bp non-templated insertion at the junction on 4p. This junction sequence was the second target selected. The third target, a deletion in chromosome 20 (p12.1p11.23), was selected since the WGS data showed an 11-bp non-templated insertion at the junction site.

The routine cytogenetic analysis had identified a reciprocal translocation t(5;7)(q35;q21) supporting a rearrangement affecting *TLX3* and *CDK6* recurrent in T-ALL (35) in patient 5. The WGS data also supported this translocation; however, as the resulting junctions were surrounded by highly repetitive sequences on 5q and 7q, the assay design was rendered unfeasible. The WGS data also identified several SVs that resulted in deletions also detected by aCGH. The majority of these, with the exception of the homozygous deletion of *CDKN2A*/B genes on 9p21.3, are not recurrent in ALL. Our final designs targeted SV junctions resulting from the deletion on 9p as well as deletions affecting 8p and 16q (**Figures S4A–C**).

For patient 6, CBA and aCGH had identified several structural events on chromosome 6 including an interstitial deletion (q14.1q15) previously described for driving T-cell leukemia progression (36), together with the recurrent deletion on chromosome 9p21.3 (including *CDKN2A/CDKN2B*). WGS data confirmed these events and revealed a *STIL*::*TAL1* fusion, a known recurrent event in T-ALL (37). The junction sequences created by these rearrangements were chosen as targets (**Figures S5A–C**).

### Whole-Genome Sequencing-Based Droplet Digital PCR Assays Detect Targets Down to a 10^−6^ Dilution

**Figure 3:**
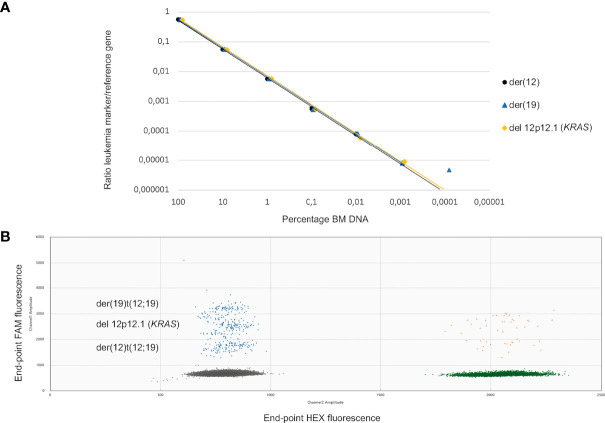
**(A, B)** Dilution series and ddPCR fluorescence plot for leukemic targets in patient 1. **(A)** Sensitivity testing for the three targets selected for patient 1, der(12), der(19) and deletion 12p. The ratios of CN target to CN reference obtained from the ddPCR assays are plotted against the percentage of gDNA from the diagnostic BM for the different dilutions using 500 ng of input gDNA. **(B)** 2D fluorescence amplitude plot generated by the QuantaSoft™ software showing the droplet clusters corresponding to the three different targets for patient 1 (der(12), der(19), and deletion 12p using 1 ng of PC in a background of 9 ng of NC. y-Axis, end-point FAM-fluorescence from targets. x-Axis, end-point HEX-fluorescence from reference gene. Negative droplets (gray). Target positive–reference negative droplets (blue). Reference positive–target negative droplets (green). Double-positive droplets (orange). ddPCR, droplet digital PCR; CN, copy number; gDNA, genomic DNA; BM, bone marrow; PC, positive control; NC, negative control.

In total, 18 ddPCR assays were designed of which three failed due to either lack of specificity or complex target regions with repetitive sequences. The 15 successful designs were further tested using serial 10-fold dilutions of diagnostic PC in NC gDNA from blood donors. No unspecific amplification was detected in DNA isolated from lymphocytes from normal healthy donors except for one target (*PAX5* in patient 2); we thus adjusted the LoD to 10^−5^ for this target. The assays were evaluated for linearity and sensitivity to determine the range where the targets were quantifiable or detectable using 500 ng of input DNA. Linearity was achieved for all assays down to a 10^−4^ dilution, and eight out of 15 assays were linear down to a 10^−5^ dilution (representative example shown in **Figure 3**). Thus, for four out of six patients, LoQ reached 10^−5^, and in the remaining two patients, the LoQ was 10^−4^. Seven of the targets were detectable one order of magnitude beyond the LoQ, and all patients had at least one target that could be detected beyond LoQ. For patients 5 and 6 (both T-ALL), 5/6 ddPCR assays had LoQ/LoD at least one order of magnitude beyond the RQ-PCR markers. The summary of LoDs and LoQs for ddPCR assays is shown in **Table 2**.

**Table 2:** ddPCR assays limit of detection (LoD) and limit of quantifiability (LoQ).

Patient	Targets	LoD	LoQ
1	der(12)t(12;19)	10^−4^	10^−4^
	der(19)t(12;19)	10^−6^	10^−5^
	12p12.1 (*KRAS*)	10^−5^	10^−5^
2	7p12.2 (*IKZF1*)	10^−6^	10^−5^
	9p13.2 (*PAX5*)	10^−5^	10^−5^
3	der8(t)(2;8)	10^−5^	10^−4^
4	4p14	10^−5^	10^−4^
	9p21.3 (*CDKN2A/B*)	10^−4^	10^−4^
	20p12.1	10^−4^	10^−4^
5	8p22	10^−5^	10^−5^
	9p21.3 (*CDKN2A/B*)	10^−5^	10^−5^
	16q22.1	10^−6^	10^−5^
6	1p3.3	10^−4^	10^−4^
	6q14.1	10^−5^	10^−4^
	9p21.3	10^−6^	10^−5^

RQ-PCR markers for patient 5, TCRB and TCRB incomplete, had a LoD/LoQ of 10^−4^/10^−3^ and 10^−4^/10^−4^, respectively. RQ-PCR markers for patient 6, TCRBB5 and TCRGV3, had a LoD/LoQ of 10^−4^ for both markers. Der, derivative chromosome; t, translocation; p, short arm; q, long arm.

### Assay Performance at Low Genomic DNA Input Levels

The assays were further tested in a multiplexed manner (1–3 targets/patient) in reactions containing low DNA input levels, i.e., 1 ng of gDNA input per well (from 10^−1^ and 10^−2^ dilutions of PC in NC). All tests were run in three replicate reactions. All targets were readily detectable at a 10^−1^ dilution containing 100 pg of PC in 900 pg of NC. At the 10^−2^ dilution (10 pg of PC in 990 pg of NC), targets for half of the patients were still detectable, while only trace signals (<3 target-positive droplets in three replicate wells) were detected for the remaining patients (**Table 3**). Moreover, the calculated CN was consistently below the theoretical CN calculated assuming no pipetting losses and 100% PCR efficiency. We concluded that the assays could accurately quantify the targets also when DNA input was minimal (**Supplementary Table 3**).

**Table 3:** Measurable residual disease assessed by the different methods.

	% MRD EoI			% MRD CB1		
	FCM/RQ-PCR	ddPCR BM ratio	ddPCR pl ratio	FCM/RQ-PCR	ddPCR BM ratio	ddPCR pl ratio
1	0.12/-	0.26	0.40	ND/-	(0.0012)	0.02
2	0.01/0.08	0.03	(0.03)	ND/NQ	0.02	(0.0004)
3	ND/-	0.01	0.03	ND/-	ND	ND
4	0.1/-	0.29	0.07	ND/-	0.03	ND
5	0.07/0.02	NA	0.04	ND/-	NA	ND
6	0.4/0.20	0.40	0.08	<0.1/NQ	NA	ND

Parentheses denote trace amounts (1–2 positive droplets). For each patient, all ddPCR targets were analyzed in a multiplexed manner, and results reflect the combined signals from all targets. MRD, measurable residual disease; BM, bone marrow; pl, plasma; FCM, flow cytometry; EoI, end of induction; CB1, end of consolidation block 1; RQ-PCR, real-time quantitative PCR; ddPCR, digital droplet PCR; NA, no sample available; NQ, detectable outside the quantifiable range; ND, not detected.

### Cell-Free DNA Yield Assessed by Droplet Digital PCR

The cfDNA level in the plasma samples was calculated from the reference gene CN in the cfDNA eluate. After correcting for initial plasma volume and cfDNA eluate input volume, this was expressed as reference gene CN/ml of plasma. Initially, some ddPCRs with cfDNA from plasma at diagnosis or relapse failed. High background fluorescence suggested that these reactions were oversaturated with cfDNA, as could be confirmed by titration of plasma volumes for cfDNA extraction, as well as cfDNA input volumes for ddPCR, until accurate cluster separation was achieved (**Figure S6**). This procedure was not required for follow-up plasma or CSF samples, which could be processed in their full volume.

**Figure 4:**
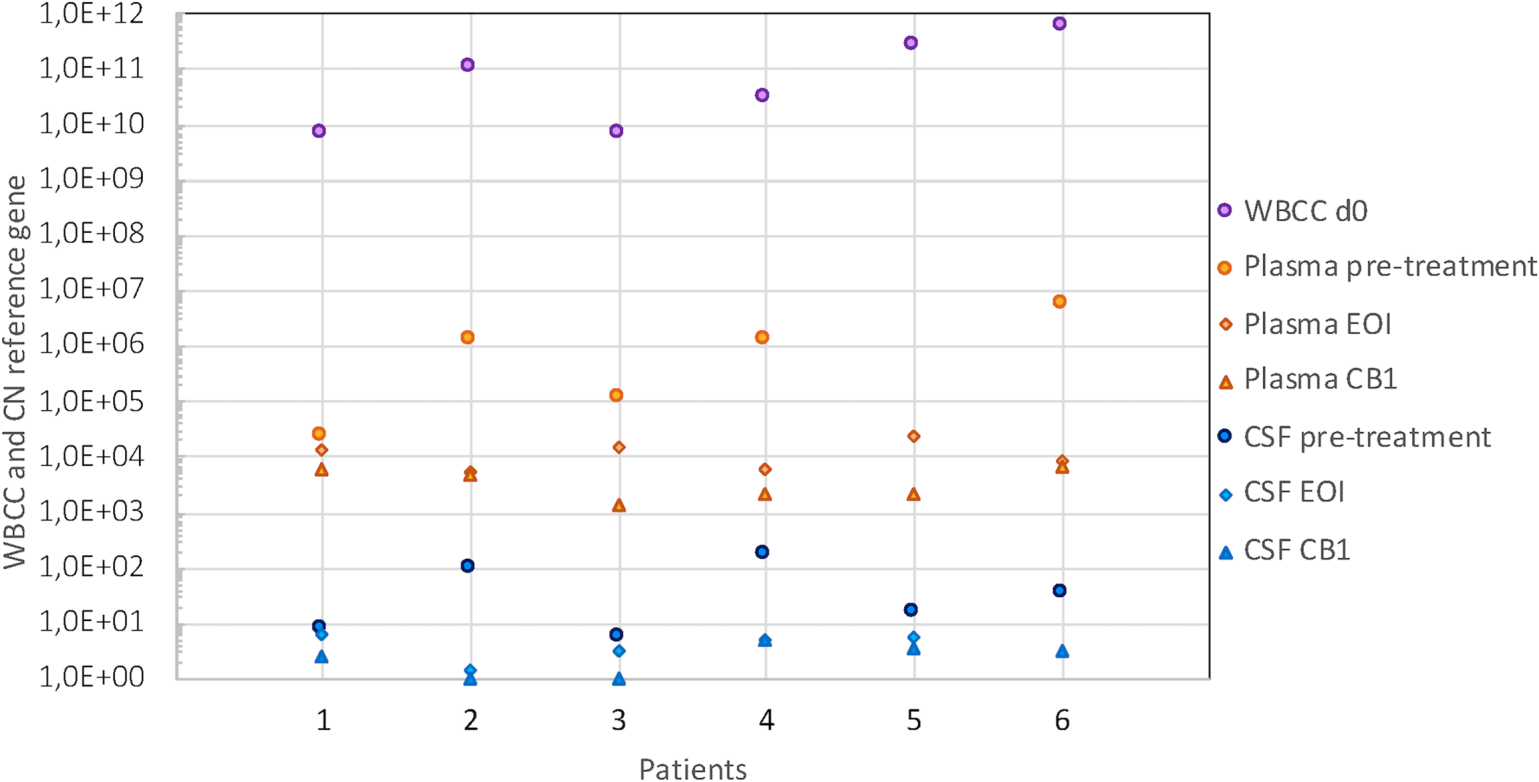
WBCC and cfDNA levels in plasma and CSF. cfDNA level expressed as CN of reference gene/ml in plasma (orange symbols) and CSF (blue symbols) in pretreatment samples (circles), at the end of induction (EoI) (diamonds) and the end of first block of consolidation therapy (CB1) (triangles). Purple circles indicate the WBCC at presentation (cells/L). The reference range for WBCC is 5.0–15.0 × 10^9^/L. WBCC, white blood cell count; cfDNA, cell-free DNA; CSF, cerebrospinal fluid; CN, copy number.

cfDNA was recovered from all plasma samples collected (n = 53), but the yields varied greatly between sampling timepoints and patients. The levels in pretreatment plasma samples differed 260-fold between cases (2.2 × 10^4^ to 5.8 × 10^6^ reference gene CN/ml) and roughly paralleled the white blood cell count (WBCC). No plasma sample at diagnosis was available for patient 5 (T-ALL), but the highest cfDNA level was seen in patient 6 (also T-ALL) with 5.8 × 10^6^ reference gene CN/ml. Overall, cfDNA levels in plasma decreased during therapy; however, the degree and timepoint varied between patients. In patients with higher CN in pretreatment plasma (>1 × 10^6^/ml), who also had the highest WBCC at diagnosis, this decrease was more pronounced during induction. By the end of the first block of consolidation therapy (CB1), cfDNA levels had dropped to levels between 1.4 × 10^3^ and 6.9 × 10^3^ reference gene CN/ml, corresponding to an approx. 0.5 to 3 log_10_ change (in patients 1 and 6, respectively) (**Figure 4**).

Patients 1, 2, and 6 showed the largest discrepancies between the kinetics of BM MRD and plasma cfDNA. These patients had transient increases in total cfDNA plasma levels during follow-up due to severe complications. For patient 1, the increase coincided with severe treatment-related pancreatitis. The increase in patient 2 was seen after sepsis and renal insufficiency. Patient 6 showed an increase concomitantly with severe treatment complications in the form of sepsis, coagulation system disorder, and renal dysfunction. Taken together, these results indicate that the total cfDNA in plasma does not only reflect the leukemic burden but is influenced by several other pathophysiological processes and therefore a less suitable MRD marker in ALL.

CfDNA was recovered from 96% of all CSF samples collected (51/53), and the amount reflected the quantity recovered from plasma for the BCP-ALL samples. In pretreatment samples (n = 6), cfDNA levels ranged between 6 and 164 reference gene CN/ml. The two follow-up samples that gave no cfDNA yield had the smallest volumes in the series (150 and 300 μl). By CB1, cfDNA levels had dropped to minimal levels (1–5 reference gene CN/ml) in all patients.

### Measurable Residual Disease Level in Bone Marrow Samples by Droplet Digital PCR

Multiplexed ddPCR assays were used to quantify MRD in the same BM samples that had been used in routine diagnostics. **Table 3** shows the MRD results by the experimental approaches and routine methods for comparison at the main timepointsused to stratify patients, EoI and end of CB1. Unfortunately, DNA follow-up samples were not available for ddPCR for patient 5 and only at EoI for patient 6. At EoI, all available BM samples that were MRD positive by routine methods (FCM and/or IG/TR RQ-PCR) were also positive by SV ddPCR. The level of MRD was in a comparable range between methods for samples with detectable residual disease, although our approach consistently detected higher values for the four patients with BCP-ALL. Patient number 3, who had undetectable MRD by FCM (RQ-PCR data not available), was positive by ddPCR. Also, patient 4, who later suffered a relapse despite being transferred to the IR group, showed a 3-fold higher MRD level with ddPCR than with FCM.

At CB1, the results were also concordant, although again for three out of five patients without detectable MRD by FCM, ddPCR showed low levels within the quantifiable range. Interestingly, patient 4 still had detectable MRD by ddPCR (0.03%) at this timepoint, while no LAIP was detected by FCM in the same sample. Taken together, the results suggest that our approach to designing molecular assays, together with ddPCR quantification, may result in a more sensitive MRD extending the range of quantifiability of leukemic targets.

We also investigated the leukemic burden measured in plasma ctDNA with the ddPCR assays at the same timepoints. The results follow the same pattern, and all but one of the samples that scored positive in BM DNA were also positive in plasma cfDNA (**Table 3**). Interestingly, for patient 1, the ctDNA results indicated a higher level of residual disease than did the BM ddPCR results.

### Kinetics of Leukemic Targets in Cell-Free DNA From Plasma by Droplet Digital PCR

**Figure 5:**
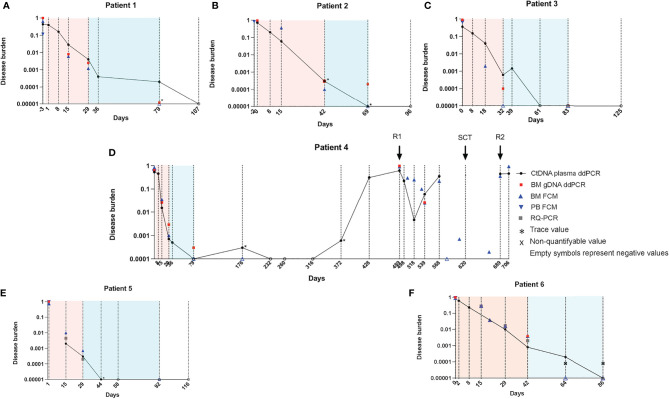
Kinetics of leukemic targets in plasma and BM by ddPCR and routine evaluation methods. Plasma ctDNA ratio by ddPCR (black circles) in patients 1-6 **(A–F)**. BM gDNA ratio by ddPCR (red squares). Asterisks denote trace amounts (<3 positive droplets). Empty symbols denote negative values. Cells with LAIP by FCM in BM (blue triangles) and in PB (inverted blue triangles). In the panels from patients 5 and 6 (both T-ALL), RQ-PCR data are shown (gray squares). Note that the lower level of detection for the RQ-PCR assay is 0.0001; RQ-PCR positive, non-quantifiable samples (black cross). BM, bone marrow; ddPCR, droplet digital PCR; ctDNA, cell-free tumor DNA; gDNA, genomic DNA; LAIP, leukemia-associated immunophenotype; FCM, flow cytometry; PB, peripheral blood; T-ALL, T-cell acute lymphoblastic leukemia; RQ-PCR, real-time quantitative PCR.

The ratio between multiplexed leukemic target CN and reference gene CN was calculated as described earlier. These ratios were plotted together with MRD information from routine evaluation during the first months following diagnosis (**Figure 5**). Overall, the level of targets decreased in ctDNA over time during induction and consolidation following the MRD level detected in BM. We found that patients 1 and 3 had higher ratios in plasma than in BM, although the difference was more pronounced by d15 than at the EoI timepoint. In contrast, patients 4, 5, and 6 cleared the targets faster in plasma than in the BM (FCM) at all timepoints investigated. Only for patient 2 did the level of the target measured in plasma shift from below the MRD in BM by d15 to above by EoI and again below BM MRD at the later timepoint.

Patient 4 suffered two relapses, and we thus investigated the kinetics of ctDNA over the entire course of the disease (**Figure 5D**). As seen for the other patients, the disease burden quickly decreased during induction, reaching no detectable level by CB1. During subsequent follow-up, only the samples taken on d176 and d372 showed trace amounts of the target. Interestingly, by d428, the plasma ratio increased markedly to 31%, 2 months prior to overt relapse (d489), which was diagnosed when the patient was admitted to the emergency room (ER)/hospital due to pain. By then, both plasma and BM showed high ratios of leukemic targets (61% and 97%, respectively). HR relapse treatment was initiated, and over the next month, the plasma ratio dropped to 0.5% (d518). Interestingly, the ratio in BM dropped to a minimum 3 weeks later (d539; 2.5%), while the plasma ratio was again increasing in the sample taken at this timepoint. Stem cell transplantation (SCT) was performed (d620), but the patient was later readmitted (d689) due to suspect post-transplantation relapse. By then, the plasma ratio was 44% and remained around this level until the last sampling timepoint, 1 month before the patient passed away.

### Detection of Leukemic Targets in Cell-Free DNA From Cerebrospinal Fluid by Droplet Digital PCR

None of the six patients had CNS involvement based on morphological examination of the CSF; however, FCM had detected cells with LAIP in patient 2. cfDNA was successfully extracted from all diagnostic CSF samples, and leukemic targets were detected in half of these, including patient 2 (**Table 4**). Target-positive CSF samples also had more copies of the reference gene than negative samples (34–164 and 6–16 reference gene CN/ml, respectively), indicating that more cells had contributed to cfDNA in those samples.

**Table 4:** CSF results at diagnosis by ddPCR and routine evaluation methods.

Patient	CSF (ml)	Target CN/ml	Ref gene CN/ml	Ratio	Cytospin	FCM % cells with LAIP
1	2	0	8		0	0
2	1	56	92	61	0	83 (<200 events)
3	2	0	6		NA	0
4	1	103	164	42	0	0
5	1	0	16		0	0
6	2	24	34	47	0	0

Parentheses denote trace amounts (1–2 positive droplets).

FCM, flow cytometry; ddPCR, droplet digital PCR; NA, no sample available; CSF, cerebrospinal fluid; CN, copy number; LAIP, leukemia-associated immunophenotype. The ratio of target molecules to reference molecules was calculated after correction for the number of targets for each patient.

**Figure 6:**
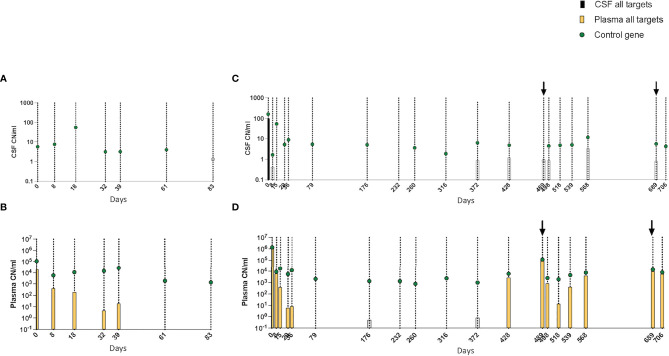
**(A–D)** Kinetics of leukemic targets in CSF and plasma by ddPCR during treatment. **(A)** CSF and **(B)** plasma results in patient 3. **(C)** CSF and **(D)** plasma results in patient 4. Empty symbols denote trace values (1–2 positive droplets). CSF, cerebrospinal fluid; ddPCR, droplet digital PCR.

Although there was a correlation between high pretreatment levels of leukemic targets in plasma and CSF positivity in some patients, this was not consistent. For example, patient 3 had few copies of the reference gene and no leukemic targets in the CSF despite having high levels in plasma at diagnosis (**Figures 6A, B**). In patient 4, who was positive for leukemic targets in the CSF at diagnosis, only trace amounts were found in later samples with no correlation to the level in the paired plasma samples (**Figures 6C, D**). Taken together, these results suggest that leukemic targets in the CSF do not solely reflect leakage from the plasma but possibly signal the presence of malignant cells inside the blood–brain barrier.

## Discussion

High-throughput technologies, in particular WGS, have shown huge potential to identify the genomic aberrations that define genetic subgroups in ALL (9) and are increasingly being introduced in the diagnostic setting for hematological malignancies (10–15). The present study was designed to explore the possibility to extend the diagnostic utility of WGS beyond genetic characterization. Since MRD assessment is the most important parameter in risk-adapted treatment protocols, we investigated the feasibility to use WGS to identify genomic structural rearrangements that involve breakage and reunion of the genome and use the novel sequences generated at the SV junctions as leukemia-specific unique markers. We hypothesized that genes recurrently rearranged in ALL are likely to be important for leukemic evolution and/or leukemia maintenance and would thus be preserved during the course of the disease and therefore constitute suitable targets to monitor treatment response.

The patients in the study were selected to challenge WGS; none of the BCP-ALL harbored the recurrent rearrangements that result in fusion genes mandatory to investigate in the NOPHO 2008 treatment protocol. Nonetheless, WGS identified suitable targets in all BCP-ALL patients and in the two T-ALL patients included. WGS enabled the detailed characterization of the breakpoints and directly provided the leukemia-specific junction sequences. Some of the chromosomal rearrangements first selected had occurred at genomic sites with highly repetitive sequences and were unsuitable as targets. This was particularly evident in patient 3, a BCP-ALL with high hyperdiploidy, where all structural events but one had occurred in such regions. This phenomenon is a known feature of the human genome: SV is enriched in regions that contain repetitive regions (38). Patient 3 was the only patient where the one suitable clonal marker was not in a genomic region reported to be involved in the pathogenesis of ALL. In order to boost the sensitivity, a single assay with double probes was designed and resulted in LoQ and LoD comparable with those of the other patients in the study. Nonetheless, this is an important limitation of the method for patients who lack a SV in a known driver gene.

The current “gold standard” for molecular monitoring of residual disease in ALL is to measure clonal IG/TR rearrangements with allele-specific quantitative PCR, a demanding method that has been extensively standardized by the EuroMRD consortium (5). In addition, this method requires a substantial amount of BM DNA and was the reason why BM DNA samples were not available from all timepoints in the patients with T-ALL. We used the criteria recommended by EuroMRD to test the performance of the SV ddPCR assays on BM. Four out of six patients’ LoQ reached 10^−5^, and in the remaining two patients, the LoQ was 10^−4^. All patients had at least one assay able to detect one leukemic cell in 100,000 normal cells, and the limiting factor for the sensitivity was the input DNA. Thus, the assays were at least as good as and potentially superior to IG/TR-PCR in regard to specificity, sensitivity, and range of quantifiability and had no background amplification from the germline. They also showed good concordance with routine MRD methods, although for a few samples, ddPCR indicated higher MRD values. This was particularly evident for patient 4, where ddPCR showed MRD above the routine methods at both EoI and CB1 timepoints, despite the fact that this patient’s assays had the lowest LoD/LoQ.

All targets selected were present in a high proportion of leukemic blasts and when possible recurrent genetic aberrations and thus likely represented truncal events. We estimated the prevalence of the targets based on filtered WGS data, and whenever possible, clinical data were also reviewed. All targets were present in >50% of cells in the diagnostic BM by WGS, clinical methods, or both. One way to improve WGS-based estimation of variant allele frequencies of the targets would be to enrich the leukemic blasts by, e.g., Ficoll separation prior to DNA extraction for WGS.

The use of several targets per patient minimizes the risk that a genetic event will be lost during clonal evolution. In order to maximize the accuracy and sensitivity when few copies of the targets are present, the assays were successfully multiplexed for all patients except in patient 3 in whom the high hyperdiploid clone only rendered one target. In all, ddPCR assays based on SVs were highly specific, which enabled precise MRD quantification with very little optimization and no need for standard curves. Hence, this WGS-based approach enabled simultaneous genetic characterization of the leukemic blasts with regard to recurrent genetic markers and identification of specific molecular targets to monitor therapy response.

The successful clinical implementation of WGS for the diagnostics of germline conditions has paved the way for the introduction of WGS in the diagnostic setting of malignancies (39, 40). In pioneering studies, WGS, often combined with WTS, has been evaluated as a diagnostic tool in acute leukemia with promising results. Compared with the standard of care multi-testing, WGS/WTS performed equally well or better in identifying clinically relevant genetic aberrations in acute leukemia patients and changed the risk classification in a proportion of cases (15, 41). Within Genomic Medicine Sweden, a national study evaluating WGS and WTS in acute leukemia diagnostics is ongoing, with the ultimate aim to replace the standard of care methods (42, 43). With decreasing sequencing costs, these types of studies will hopefully provide the required impetus to gradually implement these powerful techniques in the diagnostic setting.

A test strategy for personalized treatment protocols based on WGS is often considered premature for clinical routine due to the still relatively time-consuming methods. However, the strategy outlined in **Figure 1** is suitable for MRD testing since there is enough time to perform and analyze the WGS data and to design and evaluate different ddPCR targets before MRD monitoring is of clinical relevance.

The results also show that with our approach, MRD can be successfully measured in cfDNA from a blood sample with a sensitivity comparable to that in BM. To our knowledge, this is the first time that MRD has been monitored in cfDNA using patient-specific targets determined from WGS on BM in ALL. Using targeted ddPCR, we observed extremely high levels of ctDNA in plasma samples at diagnosis, and the kinetics showed a progressive decline during therapy. However, total cfDNA is unspecific as an MRD marker since it can be affected by other medical conditions as seen in three of our patients. On the other hand, sensitive detection and quantification of the patient-specific SV targets was possible. The selected targets decreased in plasma during the induction and initial consolidation, reflecting the leukemic burden.

The plasma markers rose from being undetectable in plasma to very high levels 2 months before an overt recurrence in the only patient in this series that suffered a relapse. This result is in line with the results from other studies that suggest that ctDNA may be a sensitive tool to monitor therapy response in hematological malignancies (44, 45) and that ctDNA not only reflects the circulating malignant cells but also represents the entire disease burden (46). Although residual leukemic cells can be detected in PB, they do not consistently represent the residual disease in the BM, and reliable MRD monitoring today calls for BM sampling (47). However, monitoring leukemia burden through plasma ctDNA in ALL could potentially be more representative than BM aspiration and has the additional advantage of being less invasive than BM sampling, which, in children, is performed under general anesthesia.

Of note, the targeted SVs could be detected in CSF from half of the patients, and 1 ml of CSF is sufficient. Our results suggest that the presence of ctDNA in CSF is not solely the consequence of leakage from the plasma but also correlates to the presence of malignant cells in the CSF. There were no traumatic punctures performed in the study, and as the CSF was collected in STRECK tubes containing cell stabilizing agents, any leukemic cells from minimal puncture bleedings are not likely to have caused false positives. The current methods for detection of CNS involvement based on morphology miss a large number of patients with leukemic cells in the CNS, and therefore, all children are treated with intrathecal prophylaxis (7, 8). Studies on FCM to improve CNS diagnostics are ongoing, and the potential contribution of ctDNA analysis on CSF also needs to be evaluated in a larger cohort.

A limitation of our approach is that SVs can occur in genomic regions harboring repetitive sequences as was the case with patient 3 with a high hyperdiploid karyotype. Despite the detection of multiple SVs in the WGS data, only one suitable ddPCR target was identified. Thus, in patients where the class defining aberration is an aneuploidy, it may be difficult to find several suitable targets and to follow MRD reliably. A limitation for all molecular methods is the turnaround time; assays need to be available by EoI to be useful for clinical decision-making. As WGS provides data for both genetic classification and SV target identification in one seamless workflow and SV ddPCR requires minimal optimization, this approach is time-efficient.

## Conclusions

WGS enabled the development of sensitive patient-specific ddPCR assays using the unique sequences generated by the chromosomal rearrangements associated with leukemia pathogenesis. The study shows that WGS not only detects the recurrent aberrations mandatory in treatment protocols but also directly identifies leukemia-specific molecular markers and that custom ddPCR assays show promising results for MRD monitoring. Moreover, the results on ctDNA suggest that monitoring the leukemia markers in plasma using ddPCR may contribute valuable information to the individualized follow-up of ALL patients in a minimally invasive manner. Further studies to systematically investigate the biology of ctDNA in ALL and its potential as an MRD marker in plasma and CSF in larger patient cohorts within current treatment protocols are warranted.

## Data Availability Statement

The datasets presented in this study can be found in online repositories. The names of the repository/repositories and accession number(s) can be found below: https://scilifelab.figshare.com/articles/dataset/WGS_for_patient_specific_MRD_in_pediatric_ALL/19678620/1

## Ethics Statement

The studies involving human participants were reviewed and approved by Ethical Review Board at Stockholm County. Written informed consent to participate in this study was provided by the participants’ legal guardian/next of kin.

## Author Contributions

CA and FR performed all experiments, interpreted the data, and wrote the manuscript. AH-S and NM collected the study samples and clinical data. LS performed the morphological examination and FCM analysis. RR and MN contributed to the study design and interpretation of results. ET and GB conceived the study, supervised the work, and contributed to manuscript writing. All authors read and approved the final manuscript.

## Funding

We are thankful for the support from the Swedish Childhood Cancer Fund through the following grants: TJ2018-0054 and TJ2021-0125, KP2018-0009, TJ 2015-0047, PR 2017-0063, and TJ2018-0012 Barncancerfonden. We would also like to thank Mary Béves forskningsfond and ALF/Region Stockholm: SLL20200306 and SLL20180046.

## Conflict of Interest

The authors declare that the research was conducted in the absence of any commercial or financial relationships that could be construed as a potential conflict of interest.

## Publisher’s Note

All claims expressed in this article are solely those of the authors and do not necessarily represent those of their affiliated organizations, or those of the publisher, the editors and the reviewers. Any product that may be evaluated in this article, or claim that may be made by its manufacturer, is not guaranteed or endorsed by the publisher.

## Abbreviations

ALL, acute lymphoblastic leukemia; aCGH, array-based comparative genomic hybridization; AML, acute myeloid leukemia; BM, bone marrow; BCP-ALL, B-cell precursor acute lymphoblastic leukemia; B-other, BCP-ALL without any of the routinely assessed classifying genetic aberrations; CB1, consolidation block 1; CBA, chromosome band analysis; cfDNA, cell-free DNA; CNA, copy number alteration; CNS, central nervous system; CR1, first complete remission; CSF, cerebrospinal fluid; cfDNA, cell-free DNA; ctDNA, cell-free tumor DNA; ddPCR, digital droplet PCR; EoI, end of induction; FCM, flow cytometry; FISH, fluorescence *in situ* hybridization; gDNA, genomic DNA; GRCh37, Genome Reference Consortium Human Build 37; HeH, high hyperdiploidy; HR, high risk; IG, immunoglobulin; IGV, Integrated Genome Viewer; IP, immunophenotype; IR, intermediate risk; LAIP, leukemia-associated immunophenotype; LoD, limit of detection; LoQ, limit of quantifiability; MRD, measurable residual disease; NA, no sample available/not applicable; NC, negative control; ND, not detected; NQ, detectable outside the quantifiable range; NIPT, non-invasive prenatal testing; NOPHO, Nordic Society for Pediatric Hematology and Oncology; NTC, no template control; PB, peripheral blood; PC, positive control; RQ-PCR, real-time quantitative PCR; SCT, stem cell transplantation; SR, standard risk; SVs, structural variants; TR, T-cell receptor; T-ALL, T-cell acute lymphoblastic leukemia; UPN, unique patient number; WBCC, white blood cell count; WGS, whole-genome sequencing.
