# Erratum: Patient-specific assays based on whole-genome sequencing data to measure residual disease in children with acute lymphoblastic leukemia: A proof of concept study

**DOI:** 10.3389/fonc.2022.1124071

**Published:** 2023-01-04

**Authors:** Frontiers Production Office

**Affiliations:** Frontiers Media SA, Lausanne, Switzerland

**Keywords:** acute lymphoblastic leukemia, liquid biopsy, disease monitoring, precision medicine, whole-genome sequencing, structural variation, technical feasibility, diagnostic performance

Due to a production error, there was a mistake in **Figure 6** as published. The part labels in the figure were not placed correctly. The corrected **Figure 6** appears below. The publisher apologizes for this mistake.

**Figure 6:**
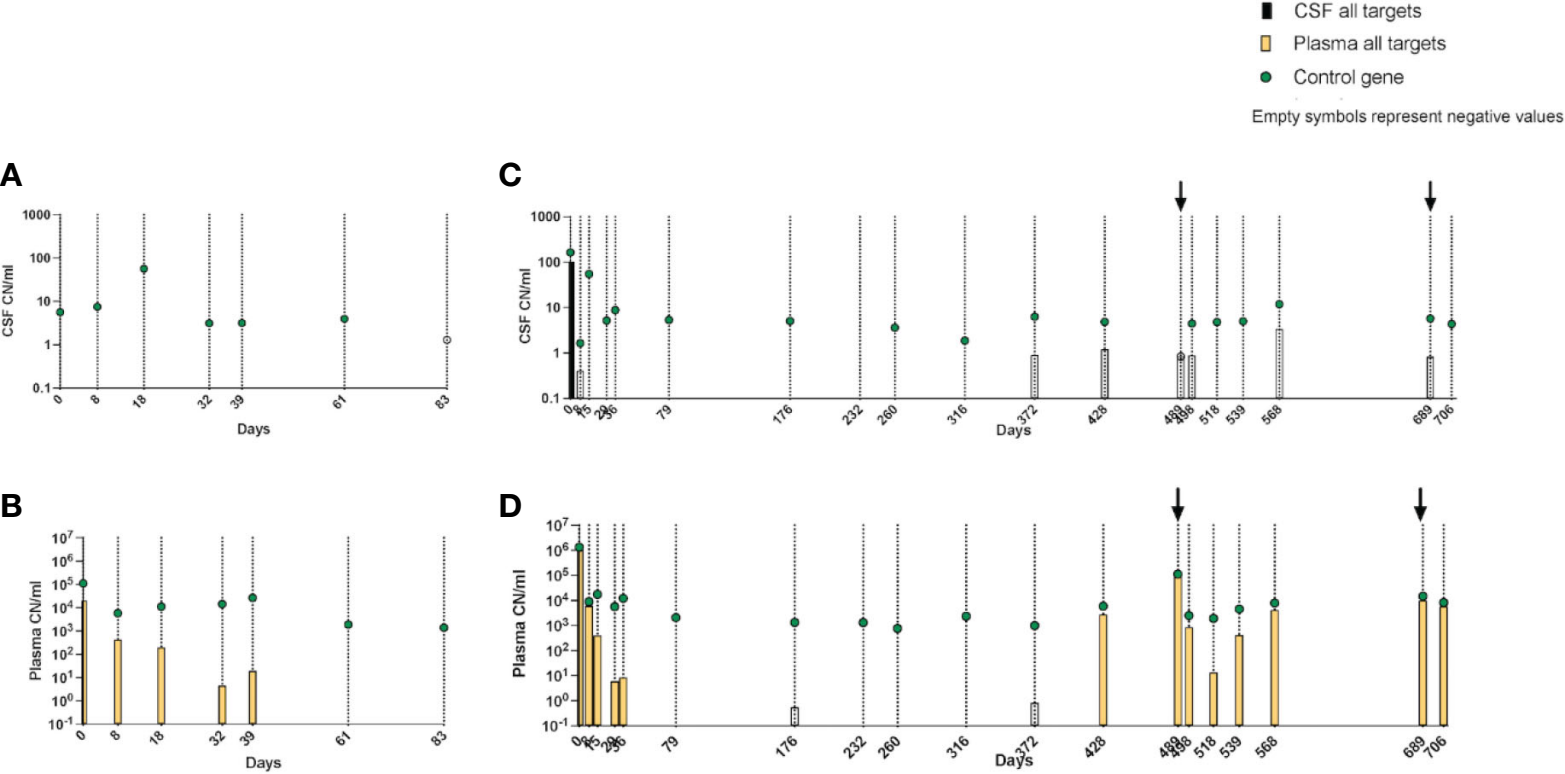
**(A–D)** Kinetics of leukemic targets in CSF and plasma by ddPCR during treatment. **(A)** CSF and **(B)** plasma results in patient 3. **(C)** CSF and **(D)** plasma results in patient 4. Empty symbols denote trace values (1–2 positive droplets). CSF, cerebrospinal fluid; ddPCR, droplet digital PCR.

The original version of this article has been updated.

