# Supplementary material for: Patient-Specific Assays Based on Whole-Genome Sequencing Data to Measure Residual Disease in Children With Acute Lymphoblastic Leukemia: A Proof of Concept Study
Source: Front Oncol. 2022 Jul 5;12:899325. doi: 10.3389/fonc.2022.899325 (PMC9296121; doi:10.3389/fonc.2022.899325)
Supplement: Supplementary file 1 [file DataSheet_1.pdf]

Chr7:50412871-50484452 (*IKZF1*)

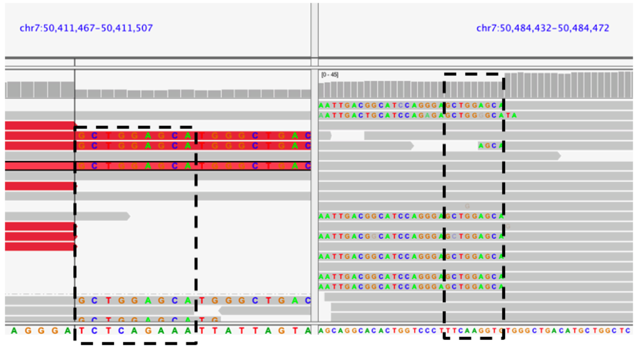

Insertion GCTGGAGCA

Fig. S1a

Chr9:36877483-37030697 (*PAX5*)

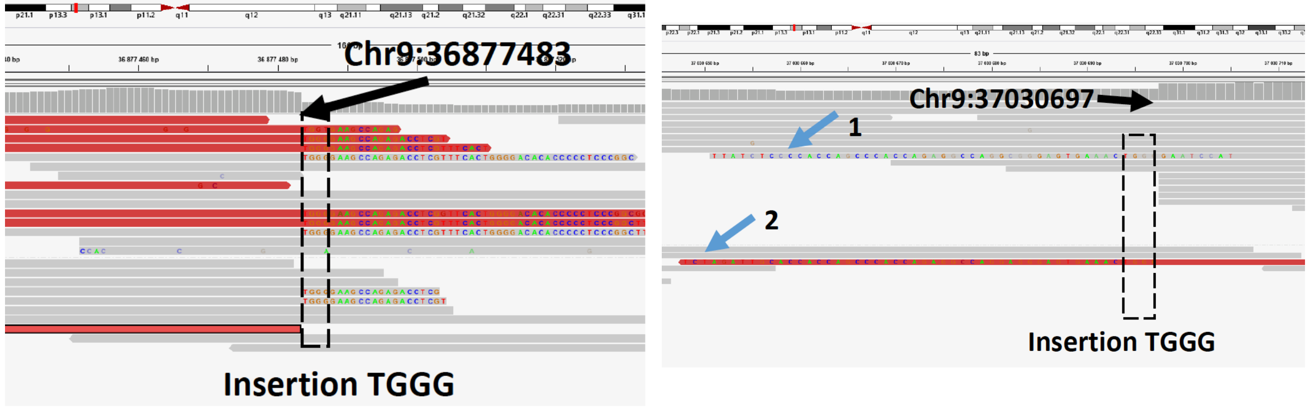

Fig. S1b

Chr6: 93,207,748

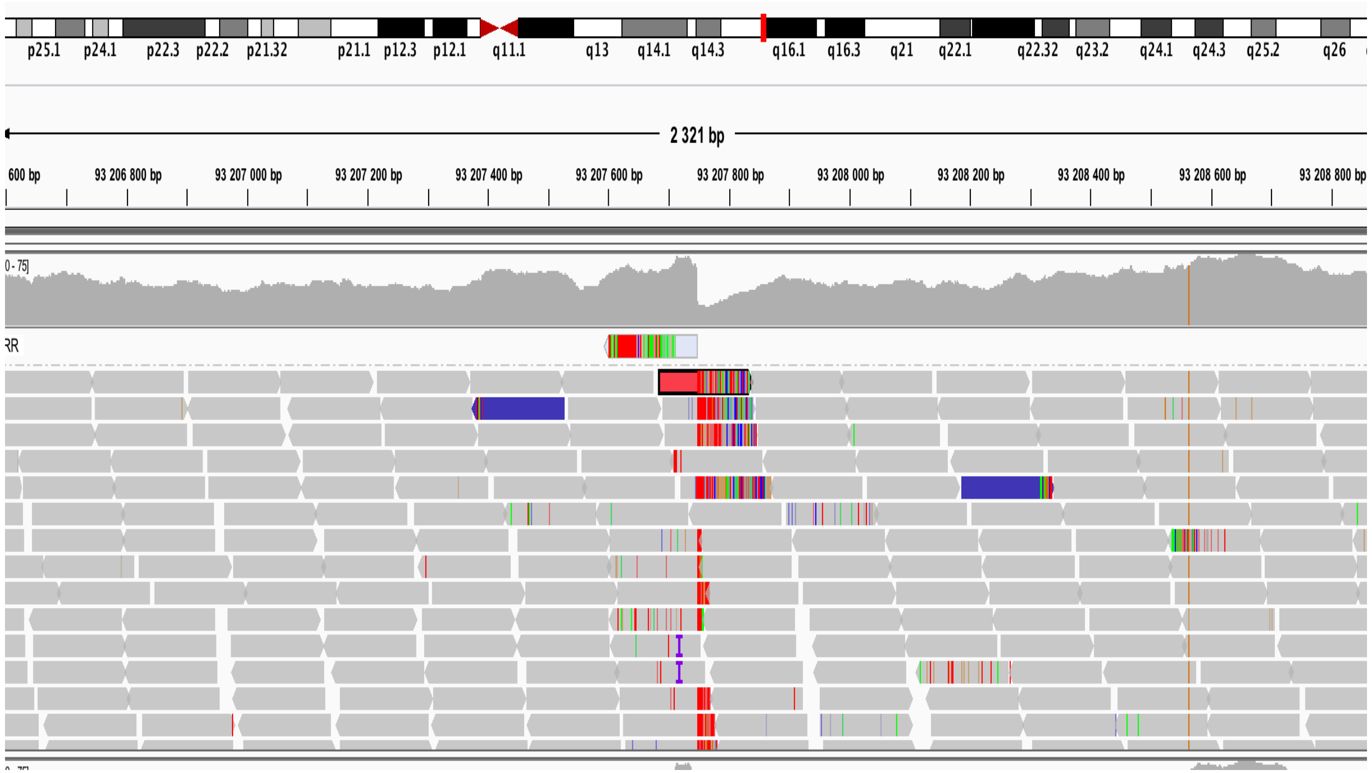

Top read:
GAAATTTTTTTTCTTTCTTTCTTTCCTTCTTTTTTGTTTTTTTTTTTTTTTTTTTTTTTTTTTTTTT
TTTTATTTATTTCTGCGTTTTTTTCGCTTTTCGAGTGCATGTGTCTCAACCTCTTTCTACAGCAT
CCTCACCTCCTCGCTCAGC

Fig. S2a

Chr2:20534566 Chr8:27199165

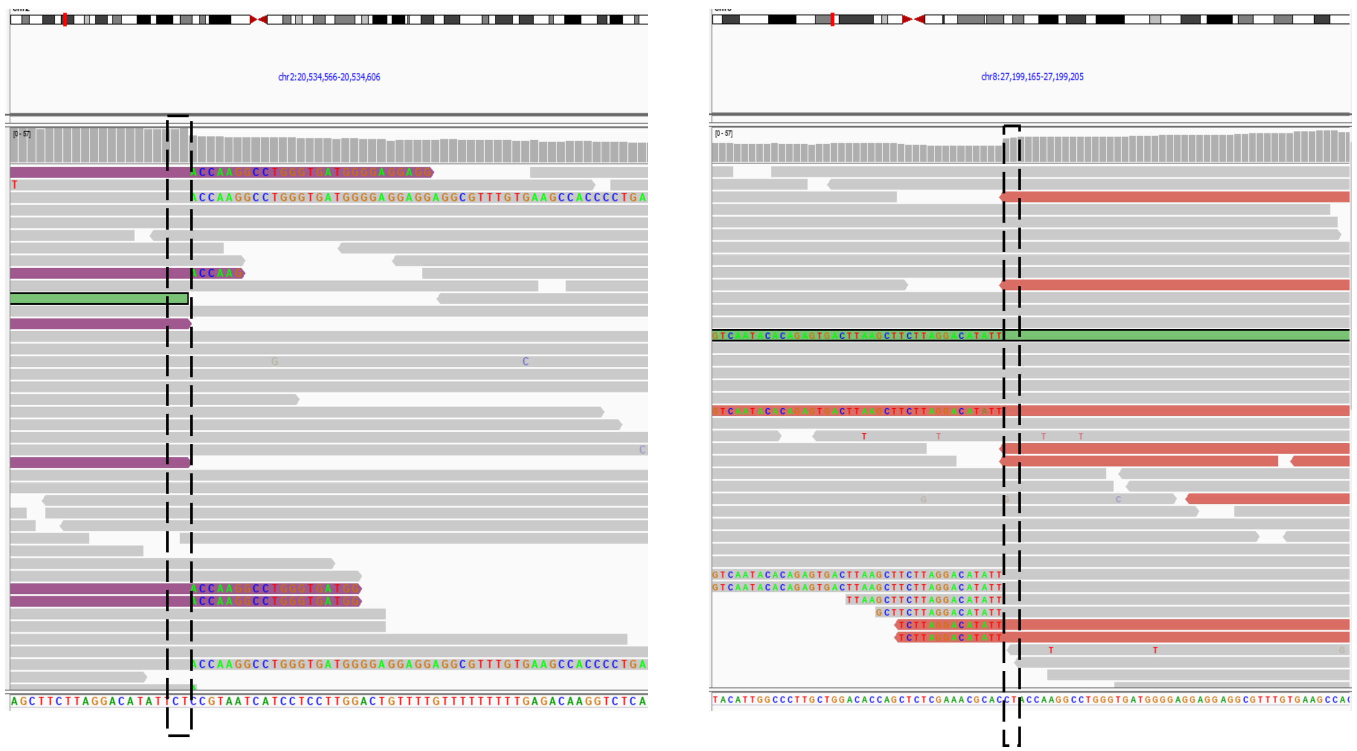

AAAACCTAGTTGAGTCACAAATAAGTCAATACACAGAGTGACTTAAGCTTCTTAGGACATATTCTACCAAGGCCTGGGTGATGGGGAGGAGGAGGCGTTTGTGAAGCCACCCC

Forward primer Forward probe Reverse probe Reverse primer

Breakpoint microhomology

Fig. S2b

(a) Chr4:34064451-72766682

Pericentric inversion and insertion: CCAGGG

(b) Chr9:5172752-34337994

Indel (delCC, insCG)

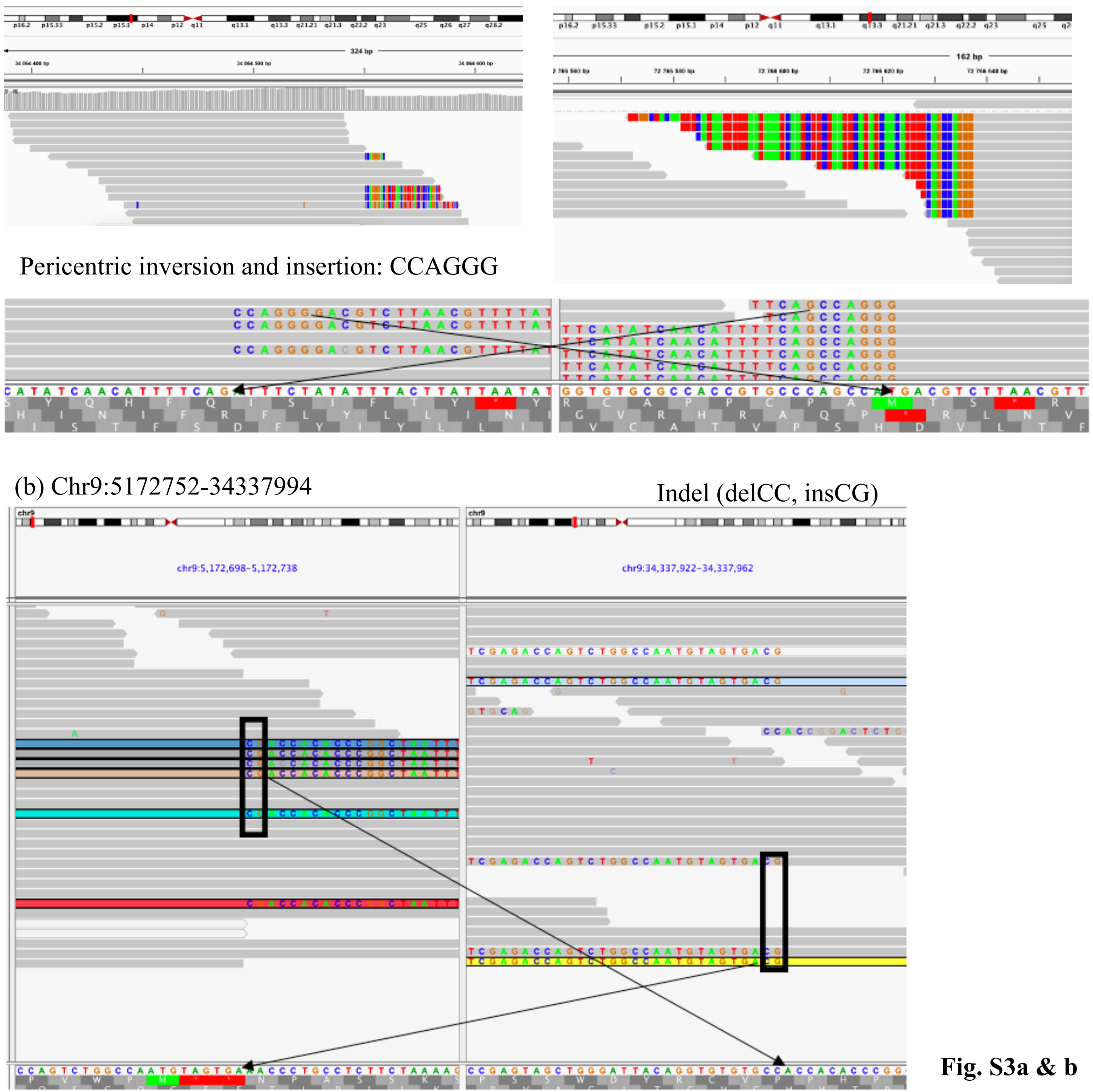

Fig. S3a & b

Chr20:11,677,759-11,678,618 (telomeric side) Chr20:21,213,451-21,215,170 (centromeric side)

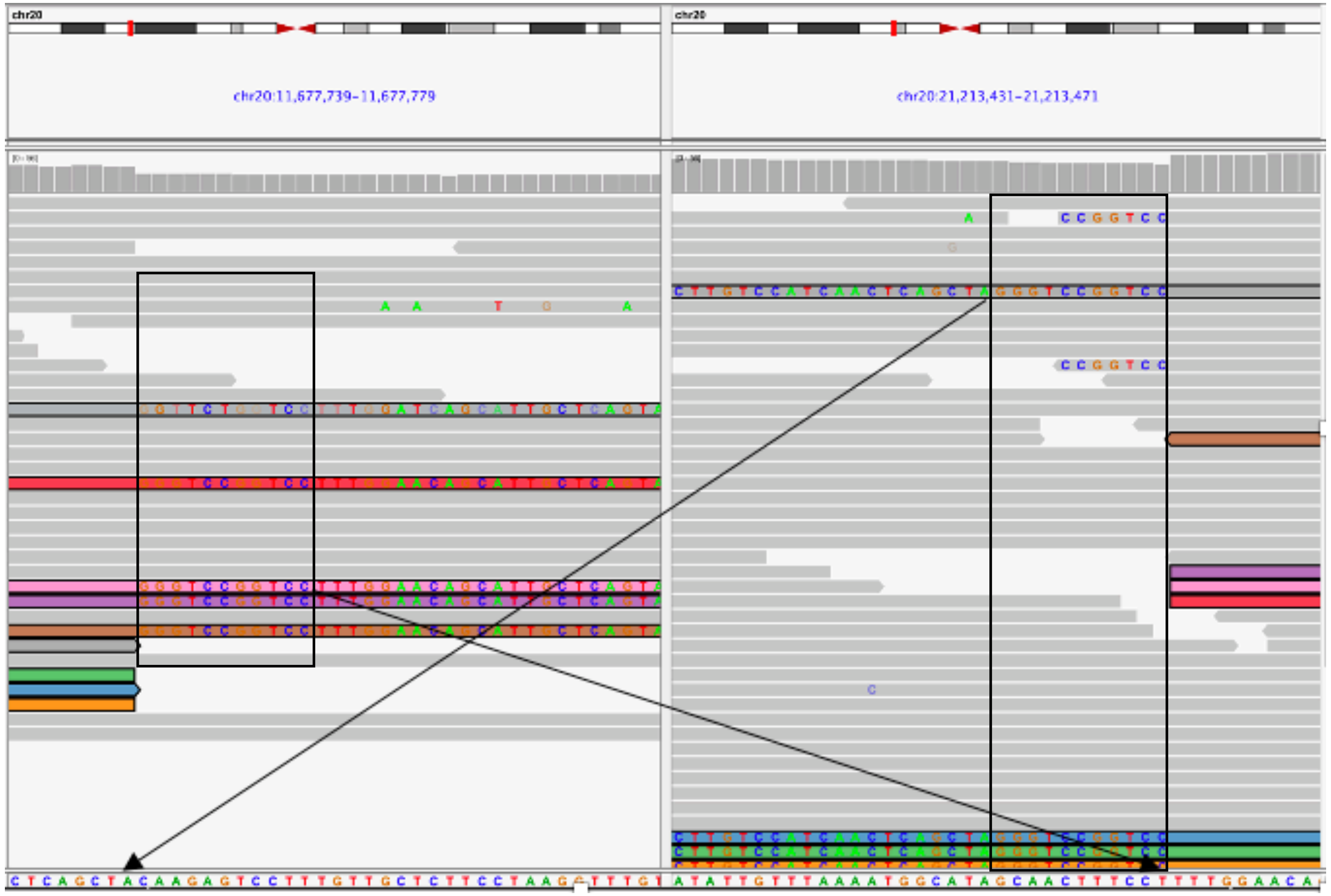

Insertion: GGGTCCGGTCC

Fig. S3c

Chr8:17 399 407-17 399 480 (telomeric side) Chr8:19 013 356-19 013 421 (centromeric side)

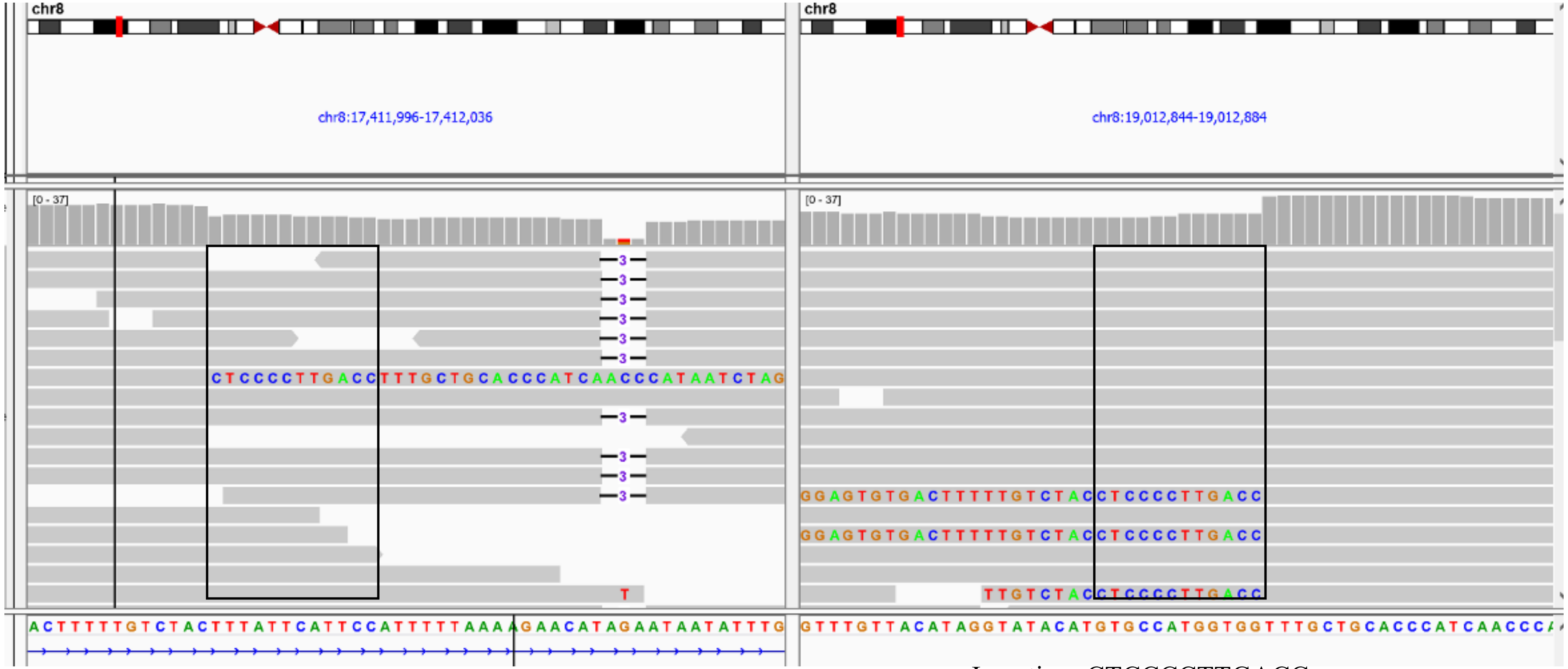

Insertion: CTCCCCTTGACC

Fig. S4a

Chr9:22 527 804-22 527 890

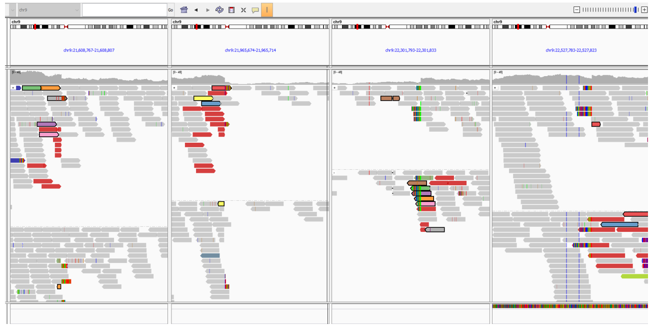

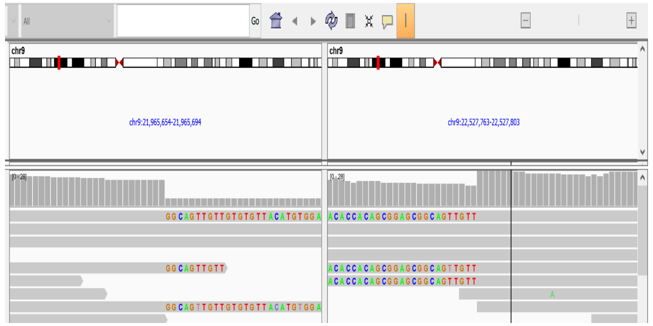

Several SVs in 9p21.3

Targeted region in 9p21.3

Fig. S4b

Fig. S4c

Chr16:67 402 941-67 403 021

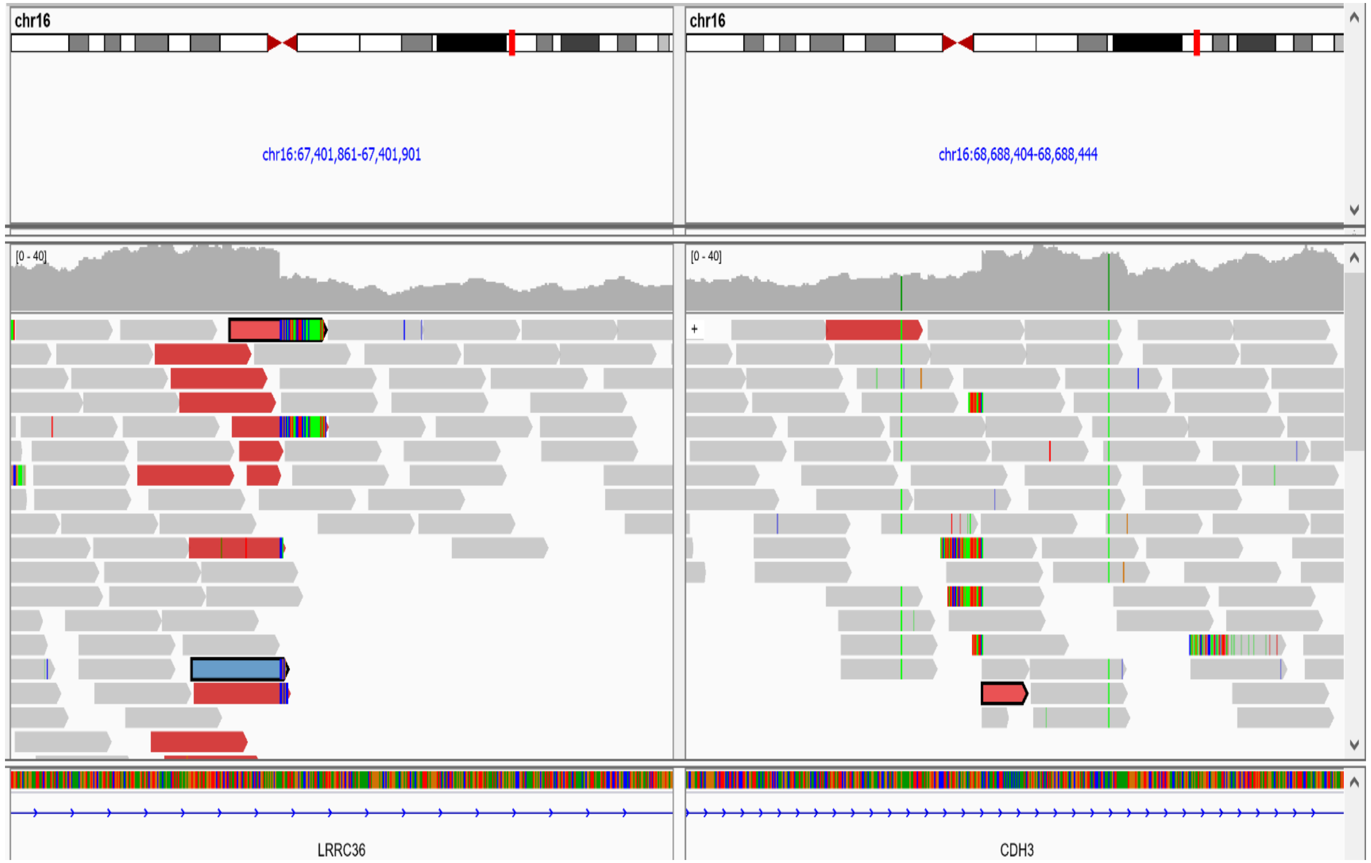

Chr1:47699244-47779335

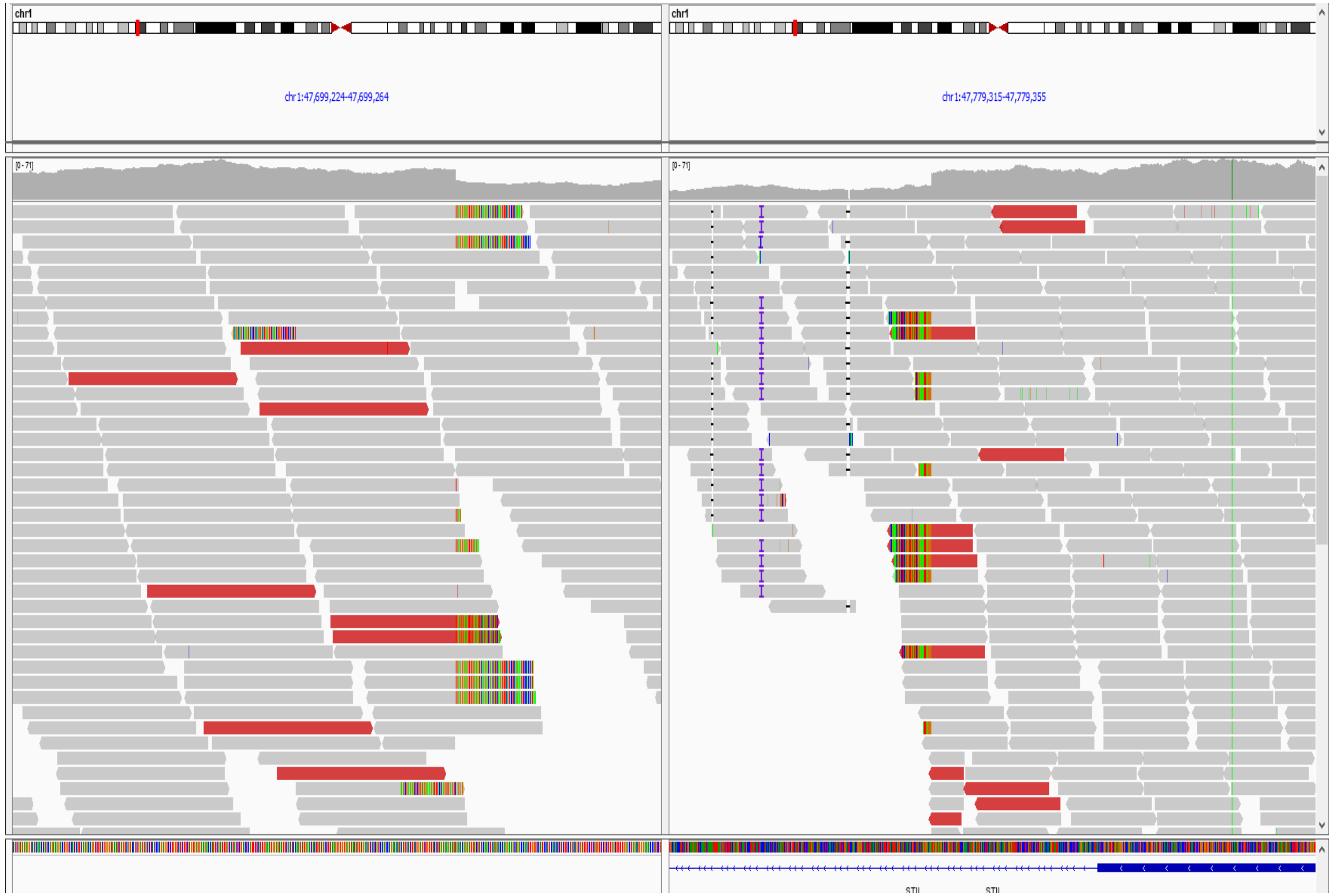

Fig. S5a

Chr6:77227792-89608335

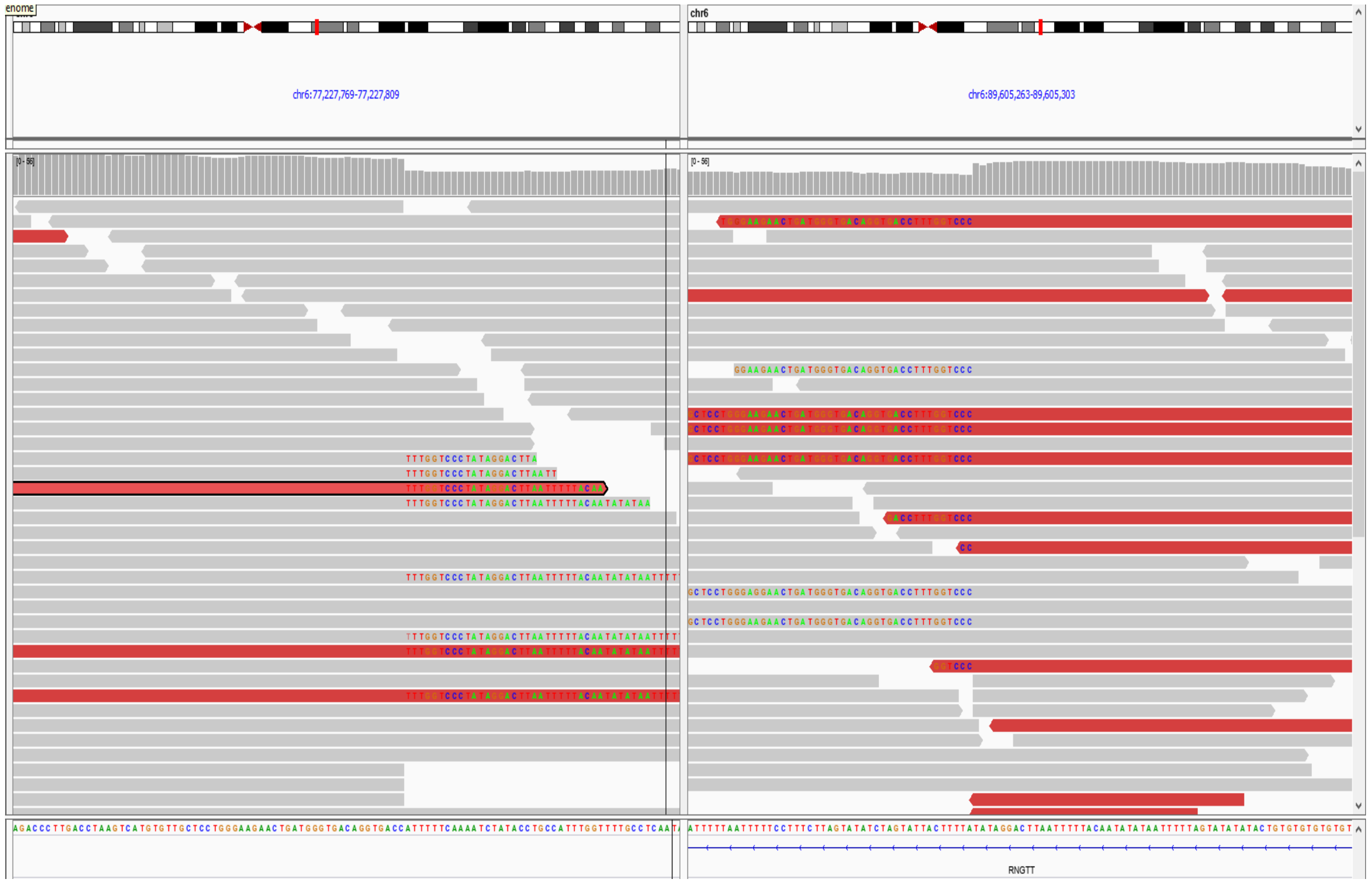

Fig. S5b

Chr9:21873676-22066963

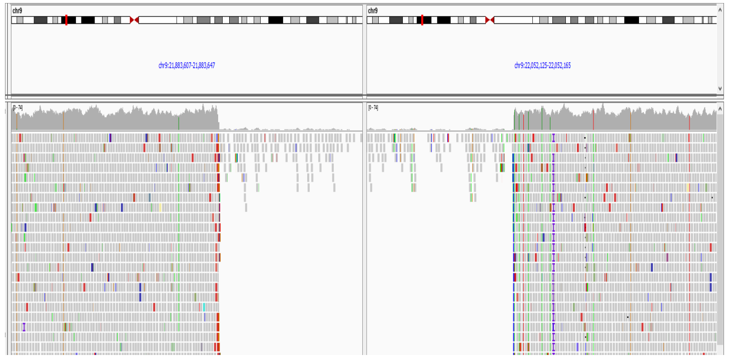

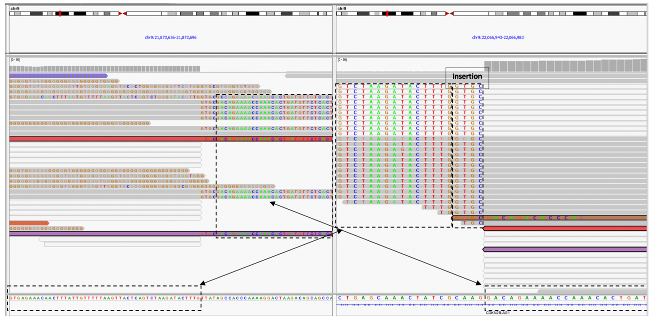

Fig. S5c
