# Supplementary material for: Patient-Specific Assays Based on Whole-Genome Sequencing Data to Measure Residual Disease in Children With Acute Lymphoblastic Leukemia: A Proof of Concept Study
Source: Front Oncol. 2022 Jul 5;12:899325. doi: 10.3389/fonc.2022.899325 (PMC9296121; doi:10.3389/fonc.2022.899325)
Supplement: Supplementary file 2 [file DataSheet_2.pdf]

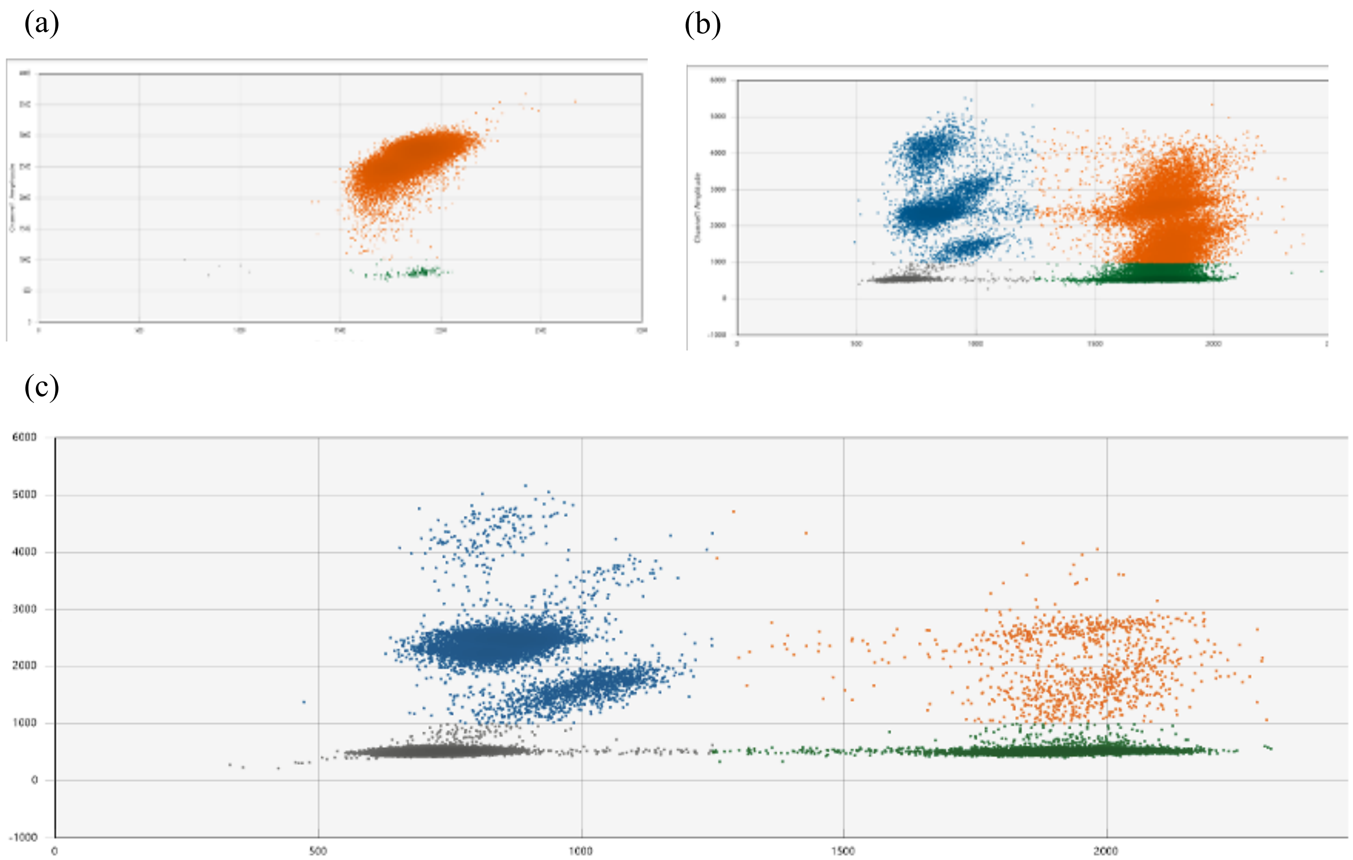

Fig. S6 ddPCR results for pre-treatment plasma sample from patient 6 (T-ALL). 2D Fluorescence amplitude plot generated by the QuantaSoft^{TM} Analysis Pro software showing the clusters corresponding to the different targets for patient 6 using decreasing input cfDNA. Plasma volumes (500, 400, 300, 200, 100, 50 and 25 ul) were diluted in PBS to a final volume of 1 ml prior to cfDNA extraction and 11 ul of eluted cfDNA was loaded in ddPCR reactions. To further optimize cluster separation ddPCR reactions were run with only 1 ul of eluted cfDNA from the 100, 50 and 25 ul dilutions. Y-axis, FAM-fluorescence from targets. X-axis HEX-fluorescence from reference gene *ABCC9*. Negative droplets (grey). Target positive/reference negative droplets (blue). Target negative/reference positive droplets (green). Double positive droplets (orange).

(a) 400 ul diagnostic plasma used for cfDNA extraction and 11 ul eluate cfDNA used for ddPCR.

(b) 25 ul diagnostic plasma used for cfDNA extraction and 11 ul eluate cfDNA used for ddPCR.

(c) 25 ul diagnostic plasma used for cfDNA extraction and 1 ul eluate cfDNA used for ddPCR.
