# Supplementary material for: Patient-Specific Assays Based on Whole-Genome Sequencing Data to Measure Residual Disease in Children With Acute Lymphoblastic Leukemia: A Proof of Concept Study
Source: Front Oncol. 2022 Jul 5;12:899325. doi: 10.3389/fonc.2022.899325 (PMC9296121; doi:10.3389/fonc.2022.899325)
Supplement: Supplementary file 3 [file Table_1.docx]

Supplementary Table 1


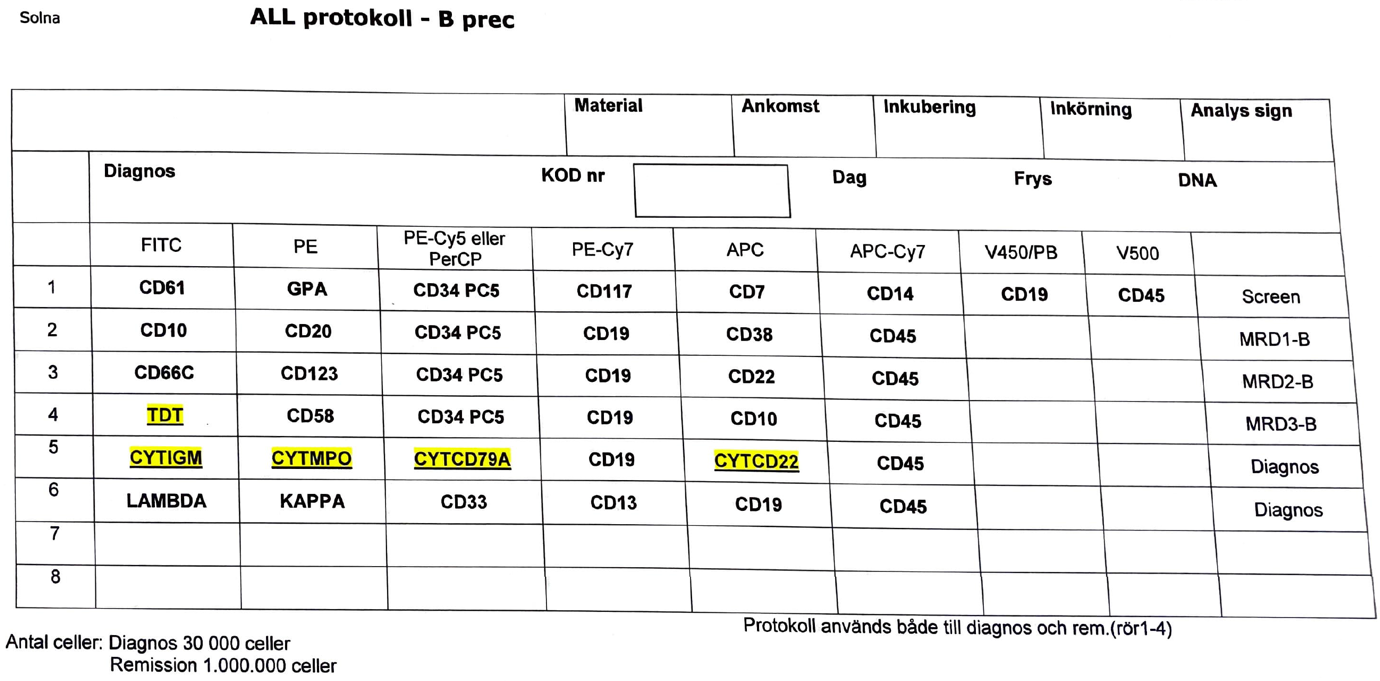


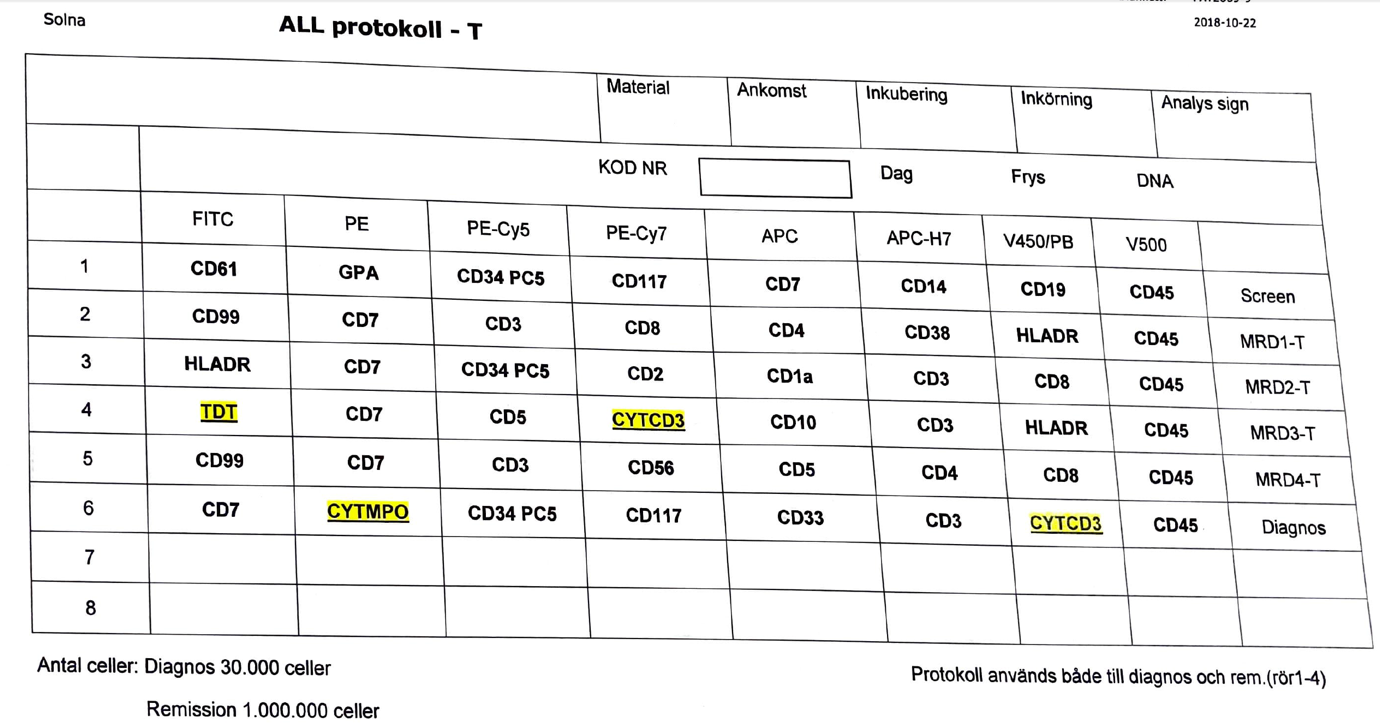


Immunophenotyping of diagnostic and follow-up samples was performed on FACSCanto/FACS Lyric flow cytometers according to the NOPHO2008 (until September 2019) and the ALLTogether protocols using standardized MRD 6- and 8-color antibody panels (all from Becton Dickinson) and the Infinicyt software program for MRD analysis. Guidelines for sample preparation and data analysis followed the NOPHO and ALLTogether laboratory guidelines. Briefly, a lyse, stain and wash method was used at the stratifying time points (day 29, day 71) and a stain, lyse and wash method for the day 15 sample.

At diagnosis, a total of 100 000 events were acquired per tube. For MRD day 15 samples up to 1 million events per MRD antibody combination were acquired to assess possible early treatment-related marker modulation and to explore day 15 response as a future prognostic marker. At least 2 MRD tubes were run for selection of the most informative marker combinations. At day 29 (TP1), day 71 (TP2) and beyond max 6 million events were acquired in total, to obtain 4 million living events excluding erythropoiesis for data analysis.

MRD data analysis was performed using Infinicyt software program. Briefly, LAIP’s of leukemic blasts are detected in the so called “empty spaces” in two-dimensional dot plots using a combination of B-cell markers according to the NOPHO2008 and ALLtogether panels.

In brief, doublets and dead cells/debris and platelet aggregates are removed using the dot plots FSC/SSC. Second, erythropoiesis is excluded using CD45/CD19 dot plots and B and T cells are gated on lineage markers (i.e. CD19 and CD7) and side scatter (SSC). Events with leukemia-associated immunophenotypes (LAIP) are identified in all plots of interest. The final MRD value is given as the mean percentage of leukemic cells of all living events excluding erythropoiesis. To be considered as MRD, a cluster of cells (>10 events) with LAIP have to be identified. A cluster of >40 events with a leukemic phenotype is considered as quantitative residual disease. If no cluster was detected, the result was termed “not detectable” or “not measurable” at a given sensitivity level.
